# Low anti-staphylococcal IgG responses in granulomatosis with polyangiitis patients despite long-term *Staphylococcus aureus* exposure

**DOI:** 10.1038/srep08188

**Published:** 2015-02-02

**Authors:** Corinna Glasner, Mirjan M. van Timmeren, Tim Stobernack, Till F. Omansen, Erwin C. Raangs, John W. Rossen, Marcus C. de Goffau, Jan P. Arends, Greetje A. Kampinga, Denny G. A. M. Koedijk, Jolanda Neef, Girbe Buist, Mehri Tavakol, Willem J. B. van Wamel, Abraham Rutgers, Coen A. Stegeman, Cees G. M. Kallenberg, Peter Heeringa, Jan Maarten van Dijl

**Affiliations:** 1 Department of Medical Microbiology, University of Groningen, University Medical Center Groningen, Hanzeplein 1P.O. Box 30001, 9700 RB Groningen, The Netherlands; 2 Department of Pathology and Medical Biology, University of Groningen, University Medical Center Groningen, Hanzeplein 1P.O. Box 30001, 9700 RB Groningen, The Netherlands; 3 Department of Medical Microbiology and Infectious Diseases, Erasmus MC, ‘s Gravendijkwal 230, 3015 CE Rotterdam, The Netherlands; 4 Department of Rheumatology and Clinical Immunology, University of Groningen, University Medical Center Groningen, Hanzeplein 1P.O. Box 30001, 9700 RB Groningen, The Netherlands; 5 Department of Internal Medicine, Division of Nephrology, University of Groningen, University Medical Center Groningen, Hanzeplein 1P.O. Box 30001, 9700 RB Groningen, The Netherlands

## Abstract

Chronic nasal carriage of the bacterium *Staphylococcus aureus* in patients with the autoimmune disease granulomatosis with polyangiitis (GPA) is a risk factor for disease relapse. To date, it was neither known whether GPA patients show similar humoral immune responses to *S. aureus* as healthy carriers, nor whether specific *S. aureus* types are associated with GPA. Therefore, this study was aimed at assessing humoral immune responses of GPA patients against *S. aureus* antigens in relation to the genetic diversity of their nasal *S. aureus* isolates. A retrospective cohort study was conducted, including 85 GPA patients and 18 healthy controls (HC). Humoral immune responses against *S. aureus* were investigated by determining serum IgG levels against 59 *S. aureus* antigens. Unexpectedly, patient sera contained lower anti-staphylococcal IgG levels than sera from HC, regardless of the patients' treatment, while total IgG levels were similar or higher. Furthermore, 210 *S. aureus* isolates obtained from GPA patients were characterized by different typing approaches. This showed that the *S. aureus* population of GPA patients is highly diverse and mirrors the general *S. aureus* population. Our combined findings imply that GPA patients are less capable of mounting a potentially protective antibody response to *S. aureus* than healthy individuals.

Granulomatosis with polyangiitis (GPA) is a systemic autoimmune disease characterized by small-vessel vasculitis and chronic necrotizing granulomatous inflammation with a predilection for the upper and lower respiratory tract and kidneys^1^. GPA is further characterized by the presence of anti-neutrophil cytoplasmic antibodies (ANCA) against proteinase 3 (PR3). Although the etiopathogenesis of GPA has been studied extensively and various genetic and environmental factors are known to contribute to inflammation, the primary cause of this disease is still debated^2^,^3^,^4^,^5^. However, upper airways infections have been repeatedly linked to GPA^2^,^3^,^6^,^7^,^8^,^9^.

Approximately 60–70% of GPA patients are chronic nasal carriers of the opportunistic pathogen *Staphylococcus aureus*, and nasal *S. aureus* carriage is associated with an increased risk of relapse^6^,^8^,^10^. Consistent with these findings, anti-bacterial treatment with co-trimoxazole reduces the risk of relapse^11^,^12^. To date, the precise mechanism by which *S. aureus* could exert a pathophysiological role in GPA has remained enigmatic. In view of the persistent activation of circulating T cells, staphylococcal superantigens (SAgs) were invoked as chronic stimuli of aberrant immune responses^13^. Indeed, it was shown that GPA patients carrying *S. aureus* positive for the superantigen toxic shock syndrome toxin-1 (TSST-1) have an increased risk for relapse, although earlier studies had not revealed a correlation between the presence of SAg genes and the expansion of specific T cell subsets in peripheral blood^14^,^15^.

*S. aureus* carriage, occurring in 20–30% of the general human population, is usually asymptomatic. However this bacterium can cause serious infections^16^. Epidemiological studies have shown that certain clonal lineages of *S. aureus* attain a geo-spatial predominance, but clear associations of specific *S. aureus* types with specific diseases have not been reported^17^,^18^,^19^. Nevertheless, it is known that virulence factors, like TSST-1 and exfoliative toxins, cause particular disease phenotypes, such as toxic shock syndrome and staphylococcal scalded skin syndrome, respectively^20^,^21^,^22^.

Information on anti-staphylococcal immune responses in GPA patients and in-depth genetic analyses of their *S. aureus* isolates have so far been lacking. Hence, it was unknown to which extent particular *S. aureus* antigens or types may contribute to GPA. To address these questions, we performed a retrospective study in 85 GPA patients. We first investigated the humoral immune response against *S. aureus* by determining serum antibody levels against a comprehensive set of *S. aureus* antigens. Subsequently, the *S. aureus* isolates were genetically characterized to investigate whether specific *S. aureus* types colonize GPA patients.

## Results

### Low levels of anti-staphylococcal antibodies in GPA patients

**Figure 1:**
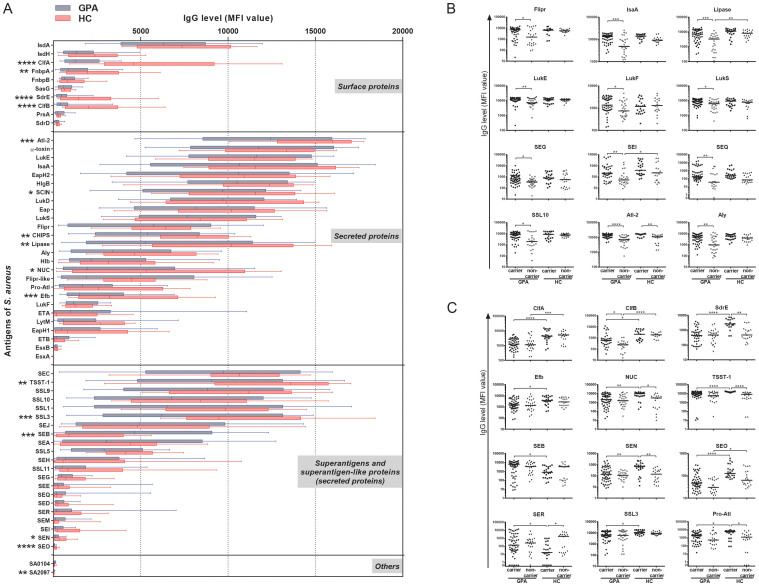
IgG responses of GPA patients and HC to staphylococcal antigens. Serum IgG levels against 59 *S. aureus* antigens were determined by bead-based Luminex flow cytometry in GPA patients, 21 *S. aureus* carriers (55 sera) and 14 non-carriers (29 sera), and HC, 10 *S. aureus* carriers (22 sera) and 8 non-carriers (20 sera). The *S. aureus* antigens comprised 10 surface proteins, 26 secreted proteins, 21 superantigens and superantigen-like proteins and 2 proteins of unknown function (indicated as others). (A) Serum IgG responses of all GPA patients (in blue) and HC (in red) to 59 *S. aureus* antigens. Depicted are the median with boxes (25% and 75%) and whiskers (10% and 90%) of all sera per group. (B) Serum IgG responses to *S. aureus* antigens that were different between *S. aureus-*carrying and non-carrying GPA patients or HC. For every indicated antigen higher responses were found in *S. aureus* carriers than non-carriers. Depicted are the individual responses and median. (C) Serum IgG responses to *S. aureus* antigens that were different between *S. aureus-*carrying GPA patients and *S. aureus-*carrying HC. Depicted are the individual responses and median. Statistical significances in A were tested using Mann Whitney-U test and in B and C using the Kruskal-Wallis test (with post-hoc Dunn's test). * p < 0.05, **p < 0.01, *** p < 0.001, and **** p < 0.0001.

Serum IgG levels against 59 *S. aureus* antigens were measured in 35 GPA patients (21 carriers, 14 non-carriers) and 18 healthy control (HC) individuals (10 carriers, 8 non-carriers) by bead-based Luminex flow cytometry. The overall antibody responses showed broad variability in both groups (Figure 1A). The highest median antibody titers were observed against several secreted proteins. In GPA patients, the IgG responses per antigen appeared overall lower than in HC, and this reached statistical significance for several surface proteins (ClfA, ClfB, FnbpA, and SdrE) and secreted proteins (Atl-2, CHIPS, Efb, Lipase, NUC, SCIN, SEN, SEO, SSL3 and TSST-1). For HC, multiple sera from different time points were measured, but serum IgG levels against *S. aureus* proteins did not change in time (data not shown). For GPA patients, two to three sera were included from the time of diagnosis, remission and/or relapse, but no differences were observed between the different disease states (data not shown). Despite the broad inter-individual variability, some clear differences were observed between *S. aureus* carriers and non-carriers in both patients and HC. As expected, overall higher responses were found in *S. aureus* carriers than non-carriers (Figure 1B). Amongst the *S. aureus* carriers, serum IgG levels against the surface proteins ClfA, ClfB and SdrE, and the secreted proteins EfB, Nuc, Pro-Atl, SEN, SEO, SSL3 and TSST-1, were lower in patients than in HC (Figure 1C) irrespective of the immunosuppressive and/or corticosteroid treatment of the patients (data not shown). Furthermore, we also measured total IgG in a subset of sera from patients and HC. This showed that the patient sera contained equal or even higher total IgG levels than HC (Table 1). Altogether, these findings show that GPA patients have lower levels of IgGs against many staphylococcal antigens than HC, irrespective of the patients' treatment.

### Detection of antigen-encoding genes in *S. aureus* isolates from GPA patients

To determine whether there is a direct connection between IgG responses to particular *S. aureus* antigens and the bacterial production of these antigens, we assessed the presence of the corresponding genes in *S. aureus* isolates from 21 of the investigated patients (75 isolates) and 10 HC (18 isolates) (Supplementary Table 5) by DNA microarray-based genotyping. The genes for the surface proteins ClfA, ClB, FnbpA and the secreted nuclease were present in all isolates from patients and HC. Interestingly, the genes for the superantigens TSST-1, SEN and SEO were less frequently detected in patient than HC isolates (5% vs 44%, 24% vs 72%, and 24% vs 72%, respectively) corresponding to the lower IgG levels against these antigens in patients, while the gene for the superantigen SEB was only detected in patient isolates (33%) corresponding to the higher IgG levels against SEB in patients. Otherwise, the *S. aureus* isolates from patients and HC had, overall, a comparable gene repertoire. Therefore, the decreased IgG responses against particular proteins (e.g. ClfA, ClfB, FnbpA) in GPA patients cannot be attributed to a lower abundance of the corresponding genes in their *S. aureus* isolates.

### The *S. aureus* population in GPA patients mirrors the general *S. aureus* population structure

**Figure 2:**
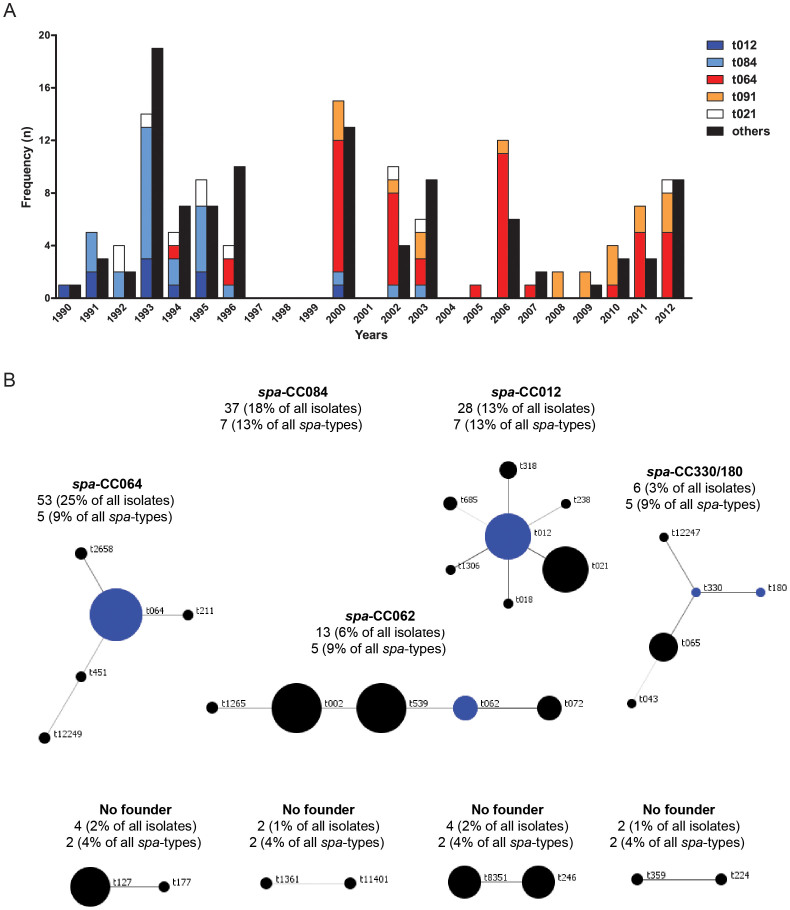
*Spa*-types of the 210 *S. aureus* isolates from GPA patients presented as (A) the five most frequent identified *spa-*types displayed by year and number and (B) *spa* clonal complexes. (A) The frequencies of the 5 predominant *spa-*types, i.e. t012 (dark blue), t084 (light blue), t064 (red), t091 (orange), t021 (white), and all other *spa-*types (black) found amongst the 210 *S. aureus* isolates from GPA patients are shown throughout the whole collection period (1990–2012). (B) The clustering of the 210 *S. aureus* isolates from GPA patients into clonal lineages was performed by BURP analysis. *spa* clonal complexes (*spa*-CCs) were composed of ≥2 related *spa*-types. A *spa-*type not clustered into any *spa*-CC was regarded as non-clonal (singleton). *spa-*types defined as founders of particular clusters are indicated in blue. The circle size is proportional to the number of isolates. The intensity of connecting lines indicates the evolutionary relationship. One hundred and forty nine isolates (71% of all isolates) were clustered in 5 *spa*-CCs (CC064, CC084, CC012, CC330/180 and CC062) and 4 groups without founder. Fifty isolates (24% of all isolates) comprising 17 *spa*-types (30% of all *spa*-types) were identified as singletons. Nine isolates (4% of all isolates) comprising two *spa*-types (t026 and t842, 4% of all *spa*-types) were excluded and two isolates (Vas103 and Vas106, 1% of all isolates) were not *spa-*typable.

The genetic diversity of the colonizing *S. aureus* isolates was determined using two complementary typing methods, namely *spa*-typing and multiple-locus variable number tandem repeat fingerprinting (MLVF)^23^. For this purpose, the *S. aureus* collection was extended to 210 isolates from 71 GPA patients. The single-locus *spa-*typing approach yielded 55 different *spa-*types, ranging in length between 3 (t026) and 14 (t328) repeats. Additionally, five novel *spa*-types were identified, and two isolates (Vas103 and Vas106) were not *spa*-typable. Thirty-one *spa*-types were represented by ≥2 isolates (184 isolates in total), while 24 *spa*-types were represented by single isolates. The most frequent *spa*-types were t064 (n = 46, 21 patients), t084 (n = 26, 16 patients), t091 (n = 19, 8 patients), t012 (n = 10, 7 patients) and t021 (n = 10, 7 patients), covering 52.4% of the investigated patient isolates. Intriguingly, the prevalence of four predominant *spa*-types showed a shift over time; t084 and t012 were solely found between 1990–2003, while t064 and t091 were predominantly found since 2000 (Figure 2A). Of the 58 patients who provided >1 *S. aureus* isolate, 39 carried isolates with different *spa-*types over time, whereas isolates from the 19 other patients showed the same *spa*-type over time. Analysis of the *S. aureus* population structure in GPA patients with the BURP algorithm revealed the respective *spa* clonal complexes (*spa*-CCs; Figure 2B)^24^. The 18 HC isolates yielded 12 different *spa*-types that partly overlapped with the *spa*-types of patient isolates (Supplementary Table 3).

**Figure 3:**
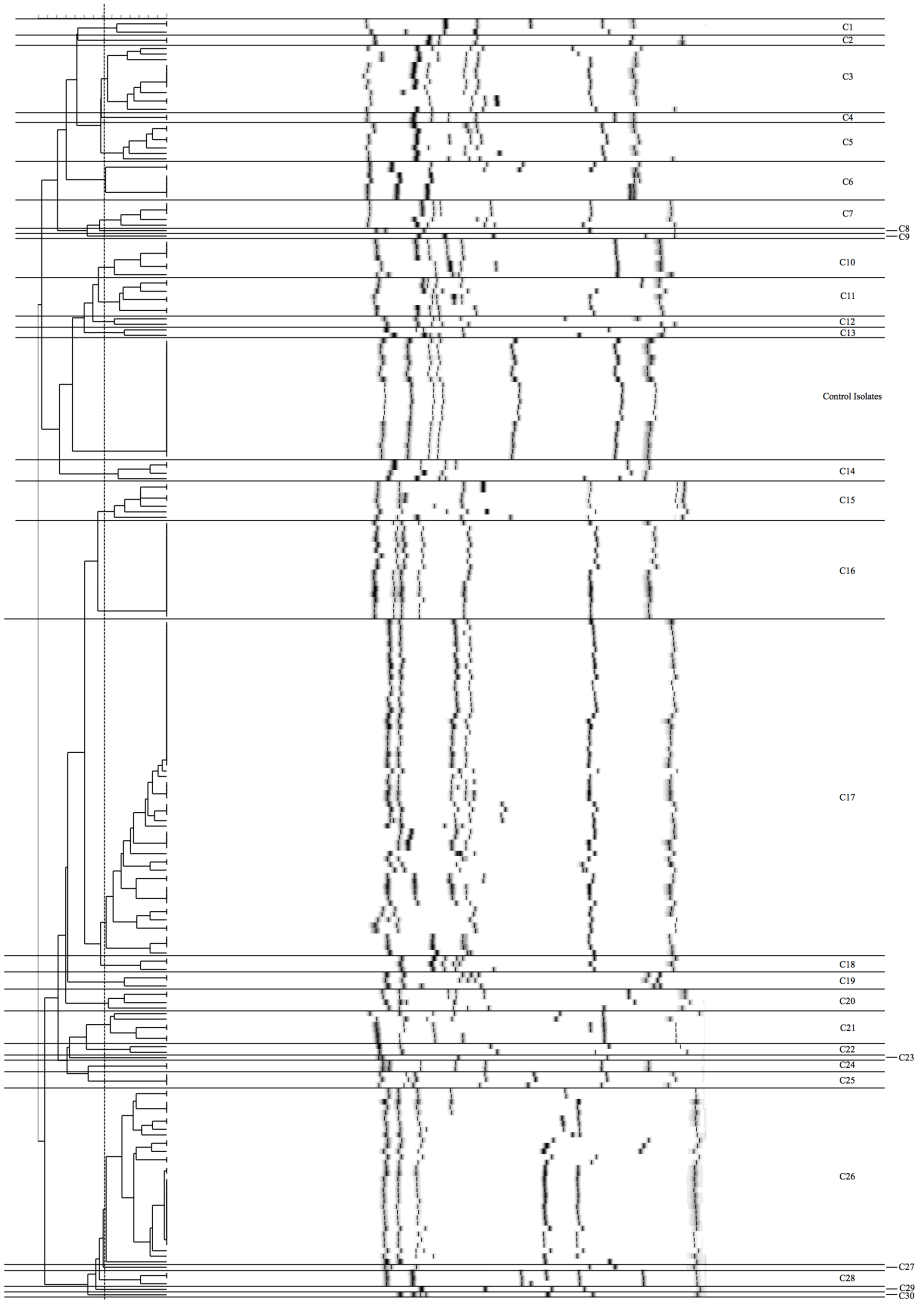
MLVF dendrogram of the 210 *S. aureus* isolates from GPA patients. An MLVF dendrogram of the 210 *S. aureus* isolates from GPA patients was generated by the UPGMA algorithm. Isolate clusters were delineated with a 66% similarity cut-off value, since this showed the highest concordance between MLVF and *spa*-typing (Adjusted Rand's Coefficient 0.671). Additionally to the 210 studied *S. aureus* isolates from GPA patients, also 22 control samples of the control isolate M2 were included in this delineation. The names of clusters are indicated at the right side of the dendrogram.

Typing of the 210 patients' isolates by MLVF identified 95 different MLVF banding patterns. Fifty-one patterns were represented by one isolate, whereas 44 patterns were represented by ≥2 isolates. Notably, two MLVF patterns were represented by 18 and 27 isolates, respectively. The highest concordance (Adjusted Rand's Coefficient 0.671) between MLVF and *spa*-typing was found with a 66% similarity cut-off value, resulting in 30 clusters (Figure 3). Six clusters contained single isolates whereas 24 clusters contained ≥2 isolates. Four clusters contained ≥12 isolates (61 isolates [C17], 32 [C26], 18 [C16] and 12 [C3]) and were derived from 27, 21, 7, and 8 patients respectively. Of the 58 patients who provided >1 isolate, 33 carried *S. aureus* belonging to different MLVF clusters over time, whereas the remaining 25 patients carried *S. aureus* belonging to the same MLVF cluster. The isolates from 18 of the latter 25 patients also had the same *spa*-type. Altogether, the combined typing data suggest that the *S. aureus* population structure in GPA patients is highly diverse, and that it has changed over time.

### Relationship between the shift in *spa*-CCs over time and antibiotic resistance profiles

**Figure 4:**
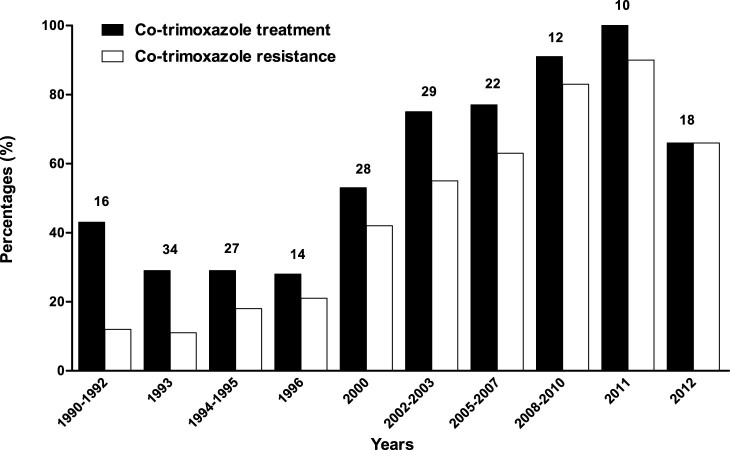
Co-trimoxazole treatment and resistance over time. The percentage of patients treated with co-trimoxazole at the time of *S. aureus* isolation in each specific period is shown in black bars. The percentage of co-trimoxazole resistant *S. aureus* isolates, determined with VITEK2 or disk diffusion, is shown in white bars. Some adjacent years of *S. aureus* collection were taken together when the single years contained only few *S. aureus* isolates. On top of the bars, the numbers of *S. aureus* isolates within that period are shown. Over time, an almost parallel increase was observed in the percentage of patients treated with co-trimoxazole at the time of *S. aureus* isolation and the percentage of co-trimoxazole resistant *S. aureus* isolates (Mann Whitney U test: p = 9 × 10^−9^ and p = 3 × 10^−12^ respectively).

The resistances to 18 different antibiotics and the antibiotic resistance genotypes of all 210 *S. aureus* isolates from GPA patients are shown in Table 3. While these isolates were susceptible to most antibiotics, resistance to penicillin, co-trimoxazole and ciprofloxacin was observed for, respectively, 72.7%, 41.4%, and 26.7% of the isolates. Notably, the *spa*-CC064 and t091 isolates collected after 2000 showed increased resistance to co-trimoxazole and ciprofloxacin compared to *spa-*CCs/*spa*-types isolated before 2000. This increased resistance seems to coincide with the increased treatment of patients with co-trimoxazole (Figure 4). Accordingly, co-trimoxazole-resistant isolates were only obtained from patients treated with this antibiotic, and co-trimoxazole-resistance was not observed for HC isolates (Table 3).

## Discussion

Although *S. aureus* carriage has been linked to relapses in GPA for many years, it had yet to be determined to which extent different *S. aureus* antigens or types could contribute to GPA. The present study was therefore undertaken to investigate the humoral immune responses of GPA patients against *S. aureus* antigens in relation to the genetic diversity of their *S. aureus* isolates. For this purpose, we studied *S. aureus* antigen-specific serum IgG levels in a large cohort of GPA patients, who were monitored for over 20 years at our hospital, in combination with extensive genotyping of their *S. aureus* isolates.

Bead-based Luminex flow cytometry of 59 *S. aureus* antigens revealed that GPA patients had circulating antibodies against many staphylococcal antigens and that antibody levels in individual patients were constant over time, irrespective of their disease state. Patients carrying *S. aureus* had overall higher anti-staphylococcal IgG levels than patients not carrying *S. aureus*, confirming previous observations^25^. The exact role of anti-staphylococcal antibodies is still debated. On the one hand, they could reflect the properties of the colonizing *S. aureus* type and/or infection episodes while, on the other hand, they could protect against colonization and/or infection. Persistent carriers of *S. aureus* have an increased risk of developing staphylococcal infections, which are in 80% of the cases caused by the endogenous strain^16^,^26^. In spite of this, persistent carriers have a lower risk of death by bacteremia compared to non-carriers^27^. This reduced risk could be the consequence of increased levels of protective antibodies against *S. aureus* that may accumulate due to long-term exposure to the colonizing strain(s)^25^,^28^. Unexpectedly, all GPA patients, irrespective of treatment with corticosteroids and/or immunosuppressives, had overall lower levels of anti-staphylococcal IgG than HC, while their total IgG levels were comparable. Moreover, we have previously shown that antibody responses following influenza vaccination in GPA patients and HC are similar, suggesting that the *S. aureus-*specific IgG response of GPA patients is aberrant^29^. The exact causes for the lower anti-staphylococcal IgG levels in GPA patients are yet unknown. Most likely, this relates to the *S. aureus*-specific immune response in GPA patients, since all patient isolates contained the genes for important host colonization factors, like ClfA, ClfB and FnbpA, against which their hosts showed lower IgG levels than HCs.

To explore the diversity of *S. aureus* carried by GPA patients, a large number of isolates sampled between 1990 and 2012 was characterized by *spa*-typing and MLVF. This revealed 55 different *spa*-types, with 5 predominant *spa*-types covering more than 50% of the isolates. The subsequent MLVF analysis revealed a considerable diversity with 95 different banding patterns. A comparison of the *spa*-types from the present collection with the Ridom *Spa* Server (October 2014) comprising 13881 different *spa*-types submitted by 107 countries with isolation dates from 2003 onwards, revealed that four of the predominant *spa-*types of our patient isolates are amongst the 20 most common *spa*-types. Additionally, a recent study on the diversity of 206 methicillin-resistant *S. aureus* isolates collected at our hospital between 2006 and 2012 revealed a similar diversity (107 MLVF banding patterns, 66 *spa*-types) as observed for our GPA isolates^30^. Taken together, these observations imply that the present GPA *S. aureus* collection mirrors the general *S. aureus* population structure after 2003.

While the Ridom *Spa* Server includes *S. aureus* typing results for different patient populations, other studies focused on particular diseases associated with increased *S. aureus* carriage rates, such as epidermolysis bullosa (EB) and cystic fibrosis (CF). A recent study from our hospital revealed that *S. aureus* isolates from EB patients were highly diverse and that these patients carried different types that fluctuated over time^31^,^32^. Surprisingly, the *spa*-types identified in the EB patient population did not overlap with the *spa*-types from the present GPA collection. A German multicenter study investigating the genetic diversity of *S. aureus* isolates from 195 CF patients identified 269 different *spa*-types among ~4000 isolates collected between 2008 and 2011 (ECCMID 2014 Abstract No. eP166). Two of the four most prevalent *spa*-types, t084 and t091, overlapped with the dominant *spa*-types in the present collection, underscoring the dominancy of these *spa*-types during the past decade.

In the present study, we have for the first time correlated *spa*-CCs/types with antibiotic resistance profiles. Although the overall antibiotic resistance of the patient isolates was very low, the abundance of co-trimoxazole-resistant isolates was higher amongst the more recent isolates, coinciding with an increase in co-trimoxazole treatment. This suggests that prolonged co-trimoxazole treatment either induced or selected for co-trimoxazole resistance. Interestingly, ciprofloxacin resistance was almost solely associated with *spa*-CC064 and mupirocin resistance with *spa*-type t091, two *spa-*CCs/types that were predominant in the later years of isolation. These associations between years of isolation, *spa-*types, antibiotics resistance and antibiotic therapy are highly relevant not only in relation to GPA patient treatment and the prevention of emerging antibiotic resistance, but also for other *S. aureus* infections. In addition, the abundance of identified co-trimoxazole-resistant *S. aureus* isolates warrants further investigations on the efficacy of prolonged co-trimoxazole treatment in GPA patients.

Superantigens, like TSST-1, cause non-specific activation of T cells resulting in polyclonal T cell proliferation and massive cytokine release^33^. Previous studies have shown that GPA patients carrying *tst-*1-positive *S. aureus* isolates have an increased risk for disease relapses^15^. Although the present study revealed only 23/210 (10.1%) *tst-*1-positive *S. aureus* isolates, all GPA patients, both carriers and non-carriers, had high IgG levels against TSST-1. This suggests that all patients encountered *tst*-1-positive *S. aureus* strains during their life. Potential associations between *S. aureus* and other autoimmune diseases, namely rheumatoid arthritis (RA) and multiple sclerosis (MS), have previously been investigated. RA patients were shown to carry different *S. aureus* types compared to HC and had higher IgG levels against TSST-1^34^. More recently, relapsing MS patients were shown to carry *S. aureus* isolates positive for the SAg gene *sea* more frequently than non-relapsing MS patients^35^. However, in the present study no apparent associations between clinical data of GPA patients and particular *S. aureus* types were found.

In conclusion, the present study investigated for the first time a large cohort of GPA patients and their *S. aureus* isolates over an extended time period. On the host side, we show that GPA patients have overall lower anti-staphylococcal IgG responses than HC. On the pathogen side, we show that GPA patients carry *S. aureus* types that are widely represented amongst the general *S. aureus* population. We therefore conclude that GPA is not associated with a particular *S. aureus* genotype, but rather with a lower ability of GPA patients to mount potentially protective antibody responses to *S. aureus*, despite their long-term exposure to this pathogen. Notably, the fact that we do not find a particular *S. aureus* type associated with GPA does not exclude a role for *S. aureus* carriage or the expression levels of particular *S. aureus* virulence factors in the GPA disease pathogenesis. We consider our findings important since they may lead to a full definition of the role of *S. aureus* in GPA. Accordingly, we believe that this lead will be relevant to the research community that investigates the role of bacterial pathogens in the onset and relapse of autoimmune diseases, and the clinicians who treat patients with such pathogen-related autoimmune diseases in general and GPA in particular.

## Methods

### GPA patients and HC

This retrospective study included 85 GPA patients (71 nasal *S. aureus* carriers and 14 non-carriers) and 18 HC (10 nasal *S. aureus* carriers and 8 non-carriers). All patients were PR3-ANCA positive, fulfilled the Chapel Hill Consensus Conference definitions for the diagnosis of GPA and regularly visited the University Medical Center Groningen (UMCG, The Netherlands)^36^. The patients were selected based on availability of stored *S. aureus* isolates and/or serum samples, but formed a representative cohort of all GPA patients from our hospital. From 21 *S. aureus-*carrying GPA patients and all 14 non-carriers serum samples from two to three different time points (diagnosis, remission, relapse) were included in the multiplex *S. aureus* antibody assay, as well as previously collected and described sera from the 18 HC^25^,^37^. From each HC at least two sera from different time points were included. Clinical data of the patients and HC, whose sera were used, are summarized in Table 1 and detailed in Supplementary Table 1. Clinical characteristics and information on the respective *S. aureus* isolates included in DNA typing from all *S. aureus-*carrying patients and HC are summarized in Table 2 and detailed in Supplementary Tables 2 and 3. This study was approved by the Medical Ethics Committee of the UMCG and conducted in accordance with the guidelines of the Declaration of Helsinki. Written informed consent was obtained from all patients.

### Multiplex *S. aureus* antibody assay

The relative amounts of serum IgGs against 59 *S. aureus* antigens were determined by bead-based Luminex flow cytometry (xMAP®, Luminex Corporation, Austin, Texas, USA) as previously described (Supplementary Table 4)^25^,^38^.

### Bacterial isolates

From 71 GPA patients, a total of 210 *S. aureus* nasal isolates (1–8 per patient, median 3) with isolation dates between 1990 and 2012 were included (Supplementary Table 3). Seventy-five of these isolates belonged to 21 patients whose anti-staphylococcal serum IgG levels were assayed. In addition, 18 *S. aureus* isolates (1–3 per HC, median 2) from the previously described HC with isolation dates between 2007 and 2012 were included as controls (Supplementary Table 3)^25^,^37^.

### *S. aureus* DNA typing

*spa*-typing and MLVF were performed as previously described^23^,^39^. To determine the clonal relatedness of the *S. aureus* population, the based upon repeat patterns (BURP) algorithm was applied (Ridom StaphType software 2.2.1)^24^.

### Antibiotic susceptibility testing

Antibiotic susceptibility was determined using the VITEK 2 system (bioMérieux, Marcy l'Etoile, France) with AST P633 cards, according to the manufacturer's protocol. The VITEK 2 minimum inhibitory concentration (MIC) results were interpreted using the VITEK 2 Advanced Expert System following EUCAST guidelines (www.eucast.org).

### DNA microarray-based genotyping

The presence of genes for staphylococcal virulence factors or antibiotic resistance in *S. aureus* isolates from patients and HC was determined with the Clondiag *S. aureus* Genotyping Kit 2.0 following the manufacturer's instructions (Alere Technologies GmbH, Jena, Germany)^40^,^41^.

### Statistical analyses

Statistical analyses were performed with GraphPad Prism (Version 6, La Jolla, California) or SPSS 20 (Chicago, USA). Differences between groups were tested for statistical significance using one-way ANOVA in case of a parametric variable and Mann-Whitney-U or the Kruskal-Wallis test in case of a non-parametric variable. A two-sided *p* value < 0.05 was considered to be statistically significant. Parametric variables are given as means ± SD. Non-parametric variables are given as median with range.

## Supplementary Material

Supplementary Information Supplementary Table 1-5

## Figures and Tables

**Table 1:** Clinical data of GPA patients and HC whose serum samples were included in the multiplex *S. aureus* antibody assay

Group	*S. aureus* carriership	No. of subjects	No. of sera	No. of male/female	Age (years) mean ± SD	No. receiving vasculitis treatment^a^/antibiotics^b^	BVAS Median (range)	Total IgG (g/L)^c^ mean ± SD
GPA	Carrier	21		11/10	51.6 ± 16.3			
Diagnosis			14			0/2	14 (4–32)	15.8 ± 5.9$
Remission^d^			21			13/16	0 (0–2)	11.0 ± 2.4
Relapse^e^			20			9/14	6 (3–16)	10.5 ± 2.9
GPA	Non-carrier	14		9/5	54.3 ± 17.4			
Diagnosis			13			4^f^/4	21 (8–28)	10.7 ± 2.1
Remission^c^			12			9/8	0 (0–0)	8.5 ± 2.1
Relapse^d^			4			2/2	14.5 (11–19)	10.1 ± 1.0
HC	Carrier	10	22	4/6	33.6 ± 11.8*	None	n.a.	11.3 ± 1.2
HC	Non-carrier	8	20	1/7	40.1 ± 12.2	None	n.a.	11.6 ± 1.2

^a^Vasculitis treatment consisted of azathioprine, (methyl) prednisolone, methotrexate, cyclophosphamide or mycophenolate mofetil.

^b^Antibiotic treatment consisted of maintenance treatment with co-trimoxazole (ranging between 0.5–3 × 960 mg daily, oral intake), in one case a patient received flucloxacilline (3 × 250 mg daily, oral intake) and in one case a patient received augmentin (2 × 1200 mg daily, oral intake).

^c^Levels of total IgG were routinely measured on a BNII nephelometer (Siemens Healthcare) in respectively 12, 10, 9, 6, 10, 3, 13 and 7 sera of the different groups.

^d^Remission serum samples were taken from GPA patients in stable remission with minimal immunosuppression, defined as: no active disease for ≥12 months, no cyclophosphamide for ≥3 months, azathioprine (or methothrexate or mycophenolate mophetil) as maintenance therapy (≤100 mg/day) in combination with prednisolone ≤10 mg/day allowed, and co-trimoxazole allowed.

^e^Relapse serum samples were taken from GPA patients irrespective of their treatment. ^f^Vasculitis treatment of these patients consisting of (methyl) prednisolone in combination with cyclophosphamide had started respectively 2, 7, 10 and 14 days before the serum sample was taken. Abbreviations: BVAS = Birmingham Vasculitis Activity Score; n.a. = not applicable.

*p < 0.05 vs GPA carrier and GPA non-carrier with one-way ANOVA followed by Tukey's post-test.

^$^p < 0.05 vs GPA carriers remission and relapse, GPA non-carriers diagnosis and remission, and HC carriers with one-way ANOVA followed by Tukey's post-test.

**Table 2:** Clinical data of GPA patients whose *S. aureus* isolates were typed

	**GPA patients (n = 71)**
No. of males (%)	40 (56.3%)
Age at inclusion, years, mean ± SD	54.7 ± 15.2
Disease duration at inclusion, years, median (range)	4.1 (0–19.9)^a^
No. with limited/generalized disease	27/44
Follow-up time, years, median (range)	11.6 (0.2–22.2)
No. of patients with relapse(s) after first isolate (%)	40 (56.3%)
No. of relapses in relapsing patients, median (range)	3 (1–13)
	***S. aureus* isolates (n = 210)**
Collection period	1990–2012
No. of obtained *S. aureus* isolates per patient: 1	13 patients
2	18 patients
3	15 patients
4	14 patients
5	8 patients
6	2 patients
8	1 patient
No. of isolates obtained during co-trimoxazole/other antibiotic treatment (%)	115 (54.8%)/9 (4.3%)
No. of isolates obtained during vasculitis treatment^b^ (%)	117 (55.7%)
No. of isolates obtained during quiescent/active disease (%)	191 (91.0%)/19 (9.0%)
BVAS in active patients, median (range)	4 (2–14)

^a^Two patients were included at the time of diagnosis of the disease.

^b^At the time of *S. aureus* sampling, 15 patients were treated with azathioprine only, 22 with prednisolone only, one with methotrexate only, 4 with cyclophosphamide only, 32 with azathioprine in combination with prednisolone, one with methotrexate in combination with prednisolone, 43 with cyclophosphamide in combination with prednisolone, and 9 patients with mycophenolate mofetil in combination with prednisolone. Abbreviations: BVAS = Birmingham Vasculitis Activity Score.

**Table 3:** Antibiotic resistance profiles of the *S. aureus* isolates from GPA patients and HC in relation to the dominant *spa*-CCs/types

*spa*-CC/type	All	*spa*-CC084	*spa*-CC012	*spa*-CC062	*spa*-CC064	t091	Others	HC
No. of isolates (%)	210^a^ (100)	37 (17.6)	28 (13.3)	13 (6.2)	53 (25.2)	19 (9.0)	60 (28.6)	18 (100)
Year of isolation, mean ± SD	ND	1995 ± 3	1996 ± 5	2003 ± 7	2005 ± 5	2007 ± 4	1999 ± 7	2008 ± 1.6
Chloramphenicol	1.5	0	0	0	6^C^	0	0	0
Ciprofloxacin	26.7	4	0	0	94	11	0	0
Clindamycin (const.)	6.1	0	4	0	9^E^	11^E^	7^E^	10
Co-trimoxazole	41.4	30	11^D^	23^d^	94	84^D^	7^d^	0
Erythromycin	10.1	0	4	42M & MP	13^E^	16E OR M	7^E^	16
Fosfomycin	0.5	0	4	0	0	0	0	0
Fusidic acid	0.5	0	0	4	4^f^	0	2^F^	0
Gentamicin	0	0	0	0	0	0	0	0
Kanamycin	0	0	0	0	0	0	0	0
Linezolid	0.5	0	0	0	2^C^	0	0	0
Mupirocin	14.2	0	11^MR^	0	21^mr^	74^MR^	0	0
Oxacillin	0	0	0	0	0	0	0	0
Penicillin	72.7	85^BL^	86^BL^	42^BL^	96^BL^	21^BL^	63^BL^	72
Rifampicin	0	0	0	0	0	0	0	0
Teicoplanin	0	0	0	0	0	0	0	0
Tetracycline	8.6	15^t^	4	42^T^	13	40	6^T^	10
Tobramycin	3.1	0	0	0	0	0	3.4^A^	0
Vancomycin	0	0	0	0	0	0	0	0

^a^For 197 *S. aureus* isolates the antibiotic resistance profile to 18 different antibiotics was determined using the VITEK2 system, and for one isolate using the standard disk diffusion assay. Twelve *S. aureus* isolates grew neither in the VITEK2 system nor in the standard disk diffusion assay; these isolates were thymidine-dependent, resulting in co-trimoxazole resistance. Numbers are percentages. The presence of antibiotic resistance genes in all 210 *S. aureus* isolates was determined by the DNA microarray system (Alere Technologies GmbH, Jena, Germany) and is indicated with letters. Abbreviations of the resistance genes: ^BL^ = β-lactamase, ^A^ = *aadD*, ^D^ = *dfrA*, ^E^ = *ermC* (& *ermB*), ^M^ = *mrsA*, ^MP^ = *mpbBM*, ^C^ = *cat*, ^T^ = *tetK*, ^F^ = Q6GD50, ^MR^ = *mupR*. Capital letters indicate that in nearly all isolates (>95%) the observed resistance phenotype is explained by the presence of the resistance gene. Small letters indicate that only in a fraction (<50%) of the isolates the resistance phenotype is explained by the resistance gene. ND = not determined.
