# Supplementary material for: Low anti-staphylococcal IgG responses in granulomatosis with polyangiitis patients despite long-term Staphylococcus aureus exposure
Source: Sci Rep. 2015 Feb 2;5:8188. doi: 10.1038/srep08188 (PMC5389034; doi:10.1038/srep08188)
Supplement: Supplementary Information — Supplementary Table 1-5 [file srep08188-s1.pdf]

Supplementary Material

Tables 1 – 5

Low anti-staphylococcal IgG responses in granulomatosis with polyangiitis patients despite long-term *Staphylococcus aureus* exposure

Corinna Glasner [1+], Mirjan M. van Timmeren [2+], Tim Stobernack [1], Till F. Omansen [1], Erwin C. Raangs[1], John W. Rossen[1], Marcus C. de Goffau[1], Jan P. Arends[1], Greetje A. Kampinga[1], Denny G.A.M. Koedijk[1], Jolanda Neef[1], Girbe Buist[1], Mehri Tavakol[3], Willem J. B. van Wamel[3], Abraham Rutgers[4], Coen A. Stegeman[5], Cees G. M. Kallenberg[4], Peter Heeringa[2#] and Jan Maarten van Dijl[1#*]

[+]These authors contributed equally.

[#]These authors contributed equally.

[1]Department of Medical Microbiology, University of Groningen, University Medical Center Groningen, Hanzeplein 1, P.O. Box 30001, 9700 RB Groningen, The Netherlands

[2]Department of Pathology and Medical Biology, University of Groningen, University Medical Center Groningen, Hanzeplein 1, P.O. Box 30001, 9700 RB Groningen, The Netherlands

[3]Department of Medical Microbiology and Infectious Diseases, Erasmus MC, ‘s Gravendijkwal 230, 3015 CE Rotterdam, The Netherlands

[4]Department of Rheumatology and Clinical Immunology, University of Groningen, University Medical Center Groningen, Hanzeplein 1, P.O. Box 30001, 9700 RB Groningen, The Netherlands

[5]Department of Internal Medicine, Division of Nephrology, University of Groningen, University Medical Center Groningen, Hanzeplein 1, P.O. Box 30001, 9700 RB Groningen, The Netherlands

Supplementary Table 1. Characteristics of GPA patients and HC whose serum samples were included in the multiplex *S. aureus* antibody assay. In this retrospective study, we included 85 GPA patients (54 with generalized and 31 with limited disease), of which 71 were nasal *S. aureus* carriers (aged 54.7±15.2 years; 40 males) and 14 were non-carriers (aged 54.3±17.4 years; 9 males), and 18 HC, of which 10 were nasal *S. aureus* carriers (aged 33.6±11.8 years, 4 males) and 8 were non-carriers (aged 40.1±12.2 years, 1 male). All patients were PR3-ANCA positive, fulfilled the Chapel Hill Consensus Conference definitions for the diagnosis of GPA and regularly visited the University Medical Center Groningen (UMCG, The Netherlands [1]. The patients were selected based on availability of stored nasal *S. aureus* isolates and/or serum samples, but formed a representative cohort of all GPA patients from our hospital. From 21 *S. aureus*-carrying GPA patients (aged 51.6±16.3 years; 11 males) and all 14 non-carriers serum samples from two to three different time points (diagnosis, remission, relapse) were included in the multiplex *S. aureus* antibody assay, as well as previously collected and described sera from the 18 HC [2,3]. From each HC at least two sera from different time points were included.

GPA and HC	Sex	Disease extent	Serum	Disease phase	Age (years)	Time since diagnosis (years)	*S. aureus* carriage	*S. aureus* positive nasal swab (yes/no)	*S. aureus* sensitivity to co-trimoxazole	Antibiotic treatment	Vasculitis treatment	Serum IgG (g/L)	Serum date
4	m	lim	1	diagnosis	70	0.0	carrier	no		-	-	13.9	29/02/00
			2	remission	73	3.6		yes	resistant	Ct	aza	9.5	08/10/03
			3	relapse	71	1.5		yes	resistant	Ct	-	11.9	23/08/01
5	m	gen	1	diagnosis	63	0.0	carrier	yes	n.a.	-	-	27.2	11/05/94
			2	remission	66	2.5		yes	n.a.	-	-	10.7	13/11/96
8	m	lim	1	remission	63	13.2	carrier	no		Ct	aza, pred	n.a.	29/02/12
			2	relapse	61	11.7		no		Ct	aza	n.a.	15/09/10
9	m	gen	1	diagnosis	79	0.0	carrier	no		-	-	n.a.	05/05/05
			2	remission	82	2.4		n.a.		-	aza, pred	n.a.	02/10/07
			3	relapse	81	1.3		n.a.		Ct	aza	n.a.	11/08/06
15	f	lim	1	remission	39	11.7	carrier	yes	sensitive	Ct	aza, pred	n.a.	16/08/00
			2	relapse	36	9.0		yes	resistant	Ct	aza, pred	n.a.	18/12/97
16	m	lim	1	remission	63	19.4	carrier.	yes	resistant	Ct	aza, pred	n.a.	21/10/09
			2	relapse	52	8.0		yes	intermediate	Ct	aza, pred	n.a.	06/05/98
18	m	lim	1	diagnosis	55	0.0	carrier	no		-	-	14.4	06/11/96
			2	remission	62	6.9		yes	n.a.	Ct	aza	n.a.	24/09/03
			3	relapse	57	2.0		no		Ct	-	n.a.	04/11/98
20	m	gen	1	remission	41	4.6	carrier	no		Ct	-	n.a.	30/10/96
			2	relapse	53	15.9		yes	sensitive	-	-	n.a.	12/03/08
21	f	gen	1	diagnosis	37	0.0	carrier	yes	sensitive	-	-	n.a.	09/02/07
			2	remission	38	1.1		no		Ct	-	n.a.	12/03/08
			3	relapse	40	3.3		yes	sensitive	-	-	n.a.	28/05/10
22	f	gen	1	remission	61	7.1	carrier	yes	n.a.	Ct	aza	n.a.	27/09/00
			2	relapse	62	7.9		no		Ct	aza	n.a.	02/08/01
23	f	gen	1	diagnosis	25	0.0	carrier	n.a.		-	-	5.1	18/05/96
			2	remission	29	4.9		yes	resistant	Ct	aza	10.6	11/04/01
			3	relapse	26	1.8		yes	intermediate	Ct	aza	6.2	11/03/98
26	f	lim	1	diagnosis	45	0.0	carrier	yes	n.a.	-	-	10.2	25/01/00
			2	remission	46	1.2		yes	n.a.	Ct	-	10.1	25/04/01
			3	relapse	48	3.2		yes	resistant	Ct	-	11.0	26/03/03
27	f	lim	1	diagnosis	33	0.1	carrier	yes	sensitive	Ct	-	13.5	27/08/03
			2	remission	39	5.8		yes	sensitive	Ct	aza	11.3	14/05/09
			3	relapse	35	2.6		yes	sensitive	Ct	cyc	12.6	16/03/06
28	m	gen	1	diagnosis	54	0.0	carrier	no		Ct	-	19.6	09/12/98
			2	remission	55	1.3		no		Ct	aza	13.6	22/03/00
			3	relapse	55	1.5		no		Ct	-	13.5	06/06/00
29	f	lim	1	diagnosis	31	0.0	carrier	yes	sensitive	-	-	15.2	19/10/00
			2	remission	40	9.0		no		Ct	aza	9.2	14/10/09
			3	relapse	32	0.6		no		-	aza	10.2	30/05/01
41	f	lim	1	diagnosis	36	0.0	carrier	n.a.		-	-	14.5	12/07/94
			2	remission	44	7.4		yes	sensitive	flu	mtx, pred	8.3	06/12/01
			3	relapse	39	3.0		no		Ct	-	10.6	03/07/97
43	m	lim	1	diagnosis	56	0.0	carrier	yes	n.a.	-	-	12.9	22/10/93
			2	remission	63	7.9		no		Ct	pred	10.4	03/10/01
			3	relapse	60	4.9		no		Ct	-	13.2	23/09/98
54	m	lim	1	remission	77	8.9	carrier	n.a.		Ct	-	n.a.	05/06/96
			2	relapse	74	5.7		n.a.		n.a.	n.a.	n.a.	11/03/93
58	m	gen	1	diagnosis	63	0.0	carrier	n.a.		-	-	20.1	02/10/92
			2	remission	67	4.1		yes	sensitive	-	-	16.6	06/11/96
			3	relapse	74	11.0		n.a.		n.a.	n.a.	5.4	15/10/03
67	f	gen	1	remission	53	7.8	carrier	no		-	-	n.a.	27/01/94
			2	relapse	50	5.7		n.a.		n.a.	n.a.	n.a.	19/12/91
71	f	gen	1	diagnosis	28	0.0	carrier	yes	n.a.	-	-	22.9	13/04/94
			2	remission	31	3.3		yes	sensitive	-	-	n.a.	17/07/97
			3	relapse	31	2.4		yes	n.a.	Ct	aza	n.a.	19/09/96
72	m	gen	1	diagnosis	57	0.0	non-carrier	n.a.		-	-	27.3	15/04/94
			2	remission	59	1.8		no		-	-	n.a.	18/01/96
73	m	gen	1	remission	73	0.5	non-carrier	n.a.		-	cyc, pred	n.a.	03/03/94
			2	relapse	75	2.5		n.a.		-	cyc	n.a.	19/03/96
74	m	lim	1	diagnosis	11	0.0	non-carrier	no		-	-	10.2	26/08/09
			2	remission	11	0.3		n.a.		Ct	-	9.1	26/11/09
75	m	lim	1	diagnosis	75	-0.1	non-carrier	no		-	-	10.3	09/01/12
			2	remission	76	1.1		no		Ct	mtx	9	05/04/13
76	m	gen	1	diagnosis	47	0.0	non-carrier	no		-	-	8.1	13/06/00
			2	remission	58	11.5		no		Ct	pred	7.7	08/12/11
77	f	gen	1	diagnosis	59	0.0	non-carrier	no		aug	cyc,methylpr	n.a.	12/03/09
			2	remission	64	4.5		no		Ct	mmf	4.7	21/08/13
			3	relapse	61	2.3		no		Ct	mtx	9.1	05/07/11
78	m	gen	1	diagnosis	45	0.0	non-carrier	no		-	-	13.8	20/03/02
			2	remission	52	6.9		no		-	-	12	18/02/09
			3	relapse	52	7.7		no		-	-	11.1	18/11/09

79	m	gen	2	diagnosis	58	0.0	non-carrier	no	-	-	n.a.	20/08/10
80	f	lim	1	diagnosis	58	0.0	non-carrier	no	-	-	9.3	15/07/10
			2	remission	60	1.6		no	Ct	aza	7.3	15/02/12
81	f	gen	1	diagnosis	60	0.0	non-carrier	no	-	-	n.a.	27/07/02
			2	remission	70	9.4		no	Ct	aza	7.9	09/12/11
			3	relapse	69	8.2		no	Ct	-	10.1	06/10/10
82	m	gen	1	diagnosis	34	0.1	non-carrier	no	Ct	cyc, pred	n.a.	03/05/07
			2	remission	35	1.5		no	Ct	aza	9.5	18/09/08
83	f	gen	1	diagnosis	51	0.0	non-carrier	no	-	-	12.5	01/05/12
84	f	gen	1	diagnosis	73	0.0	non-carrier	no	Ct	cyc, pred	n.a.	16/01/06
			2	remission	76	2.6		no	-	mmf	10.6	01/08/08
85	m	lim	1	diagnosis	59	0.0	non-carrier	no	Ct	cyc, pred	n.a.	08/08/08
			2	remission	60	1.4		no	Ct	aza, pred	6.8	06/01/10
HC1	f		1		40		carrier		-	-	12.2	01/06/07
			2		43				-	-	10.8	01/04/10
HC2	m		1		23		carrier		-	-	n.a.	01/06/07
			2		23.5				-	-	n.a.	01/12/07
			3		26				-	-	12.9	01/04/10
HC3	m		1		42		carrier		-	-	n.a.	01/06/07
			2		42.5				-	-	n.a.	01/12/07
			3		45				-	-	13.5	01/04/10
HC4	f		1		32		carrier		-	-	n.a.	01/06/07
			2		32.5				-	-	11.2	01/12/07
			3		35				-	-	11.3	01/04/10
HC5	f		1		53		non-carrier		-	-	n.a.	01/06/07
			2		53.5				-	-	n.a.	01/12/07
			3		56				-	-	11	01/04/10
HC6	m		1		53		non-carrier		-	-	n.a.	01/06/07
			2		53.5				-	-	n.a.	01/12/07
			3		56				-	-	13.1	01/04/10
			4		58				-	-	12.9	01/07/12
HC7	f		1		53		non-carrier		-	-	n.a.	01/06/07
			2		54				-	-	11.3	01/02/08
			3		58				-	-	10.7	01/07/12
HC8	m		1		25		carrier		-	-	9.4	01/04/10
			2		27				-	-	10.1	01/07/12
HC9	f		1		26		carrier		-	-	10.9	01/04/10
			2		28				-	-	9.9	01/07/12
HC10	m		1		48		carrier		-	-	n.a.	01/06/07
			2		49				-	-	11.5	01/02/08
HC11	f		1		29		carrier		-	-	n.a.	01/06/07
			2		30				-	-	12.5	01/02/08
HC12	f		1		46		non-carrier		-	-	n.a.	01/06/07
			2		47				-	-	12.1	01/02/08
HC13	f		1		27		non-carrier		-	-	n.a.	01/06/07
			2		28				-	-	9.9	01/02/08
HC14	f		1		30		carrier		-	-	n.a.	01/06/07
			2		31				-	-	10.7	01/02/08
HC15	f		1		41		carrier		-	-	n.a.	01/06/07
			2		41.5				-	-	n.a.	01/12/07
HC16	f		1		30		non-carrier		-	-	n.a.	01/06/07
			2		30.5				-	-	n.a.	01/12/07
HC17	f		1		27		non-carrier		-	-	n.a.	01/06/07
			2		27.5				-	-	n.a.	01/12/07
HC18	f		1		32		non-carrier		-	-	n.a.	01/06/07
			2		32.5				-	-	n.a.	01/12/07

Abbreviations: HC = healthy control; lim = limited disease (upper and/or lower airway involvement not accompanied by glomerulonephritis); gen = generalized disease (including kidney involvement); Ct = co-trimoxazole; flu = flucloxacillin; aug = augmentin; aza = azathioprine; pred = prednisolone; cyc = cyclophosphamide; mtx = methotrexate; methylpred = methylprednisolone; - = no treatment; n.a. = not available.

References

1. Jennette, J. C. *et al.* 2012 Revised international chapel hill consensus conference nomenclature of vasculitides. *Arthritis Rheum* **65,** 1–11 (2013).
2. Verkaik, N. J. *et al.* Anti-staphylococcal humoral immune response in persistent nasal carriers and noncarriers of *Staphylococcus aureus*. *J Infect Dis* **199,** 625–632 (2009).
3. Verkaik, N. J. *et al.* Immune evasion cluster-positive bacteriophages are highly prevalent among human *Staphylococcus aureus* strains, but they are not essential in the first stages of nasal colonization. *Clin. Microbiol. Infec.* **17,** 343–348 (2011).

Supplementary Table 2. Characteristics of GPA patients whose *S. aureus* isolates were typed. From 71 GPA patients, a total of 210 *S. aureus* nasal isolates (1-8 per patient, median 3) with isolation dates between 1990 and 2012 were included in *S. aureus* DNA typing. Clinical data from the time of *S. aureus* isolation was retrieved from the patients' medical record. The number of diseases relapses occurring after *S. aureus* isolation was retrieved from the patients' medical records until the patients' death or December 31, 2012.

Patient number	Sex	Disease extent	Organ involvement at the time of diagnosis	Isolate	Age (years)	Time since diagnosis (years)	Follow up of medical record (years)	No. of relapses	Disease activity	Antibiotic treatment	Vasculitis treatment	Sample	Co-trimoxazole phenotype
1	m	lim	ent, lung, joints	1	32	0.0	10.9	4	a (9)	Ct	-	Vas146	S
				2	36	4.1	6.8	1	a (12)	Ct	mmf, pred	Vas001	S
				3	43	10.7	0.2	0	q	Ct	aza	Vas190	R
2	m	gen	ent, lung, kidney, skin	1	60	0.4	10.8	2	q	Ct	-	Vas151	R
				2	62	2.3	8.9	1	q	Ct	aza	Vas174	R
				3	64	4.4	6.8	0	q	Ct	aza, pred	Vas002	R
				4	70	10.3	0.9	0	q	Ct	aza, pred	Vas003	R
3	m	gen	ent, lung, kidney, skin	1	37	0.1	6.8	0	q	Ct	cyc, pred	Vas004	R
				2	37	0.3	6.6	0	q	Ct	cyc, pred	Vas005	R
				3	37	0.4	6.5	0	q	Ct	cyc, pred	Vas006	R
4*	m	lim	ent	1	76	6.1	4.9	0	q	-	-	Vas007	R
5*	m	gen	ent, kidney, skin, neuro,	1	66	2.3	16.3	0	q	-	-	Vas052	S
			bowel	2	66	2.5	16.0	0	q	-	-	Vas053	S
				3	71	7.7	10.8	0	q	-	-	Vas149	R
				4	75	11.9	6.6	0	q	-	-	Vas008	S
6	m	gen	ent, lung, kidney	1	62	0.8	6.5	0	q	-	aza, pred	Vas009	S
7	m	gen	ent, lung, kidney, joints	1	53	5.3	10.4	2	q	Ct	aza, pred	Vas158	S
				2	57	9.5	6.2	0	q	Ct	cyc, pred	Vas010	R
8*	m	lim	ent	1	51	1.9	12.0	5	q	Ct	mtx, pred	Vas222	R
				2	57	7.3	6.6	1	q	Ct	cyc, pred	Vas011	R
				3	57	7.8	6.2	1	q	Ct	cyc, pred	Vas012	R
				4	61	11.9	2.0	0	q	Ct	aza, pred	Vas013	R
				5	62	12.1	1.8	0	q	Ct	aza, pred	Vas014	R
				6	63	13.1	0.8	0	q	Ct	aza, pred	Vas178	R
9*	m	gen	lung, kidney, neuro	1	80	0.3	2.7	1	q	Ct	cyc, pred	Vas175	R
				2	80	1.0	2.1	1	q	Ct	aza, pred	Vas015	R
				3	81	1.4	1.7	0	q	Ct	pred	Vas016	R
				4	81	1.4	1.7	0	q	Ct	pred	Vas017	R
10	m	lim	ent	1	53	1.4	6.5	1	a (6)	-	cyc, pred	Vas018	S
				2	53	1.6	6.3	1	q	Ct	cyc, pred	Vas019	S
				3	54	2.0	5.9	1	q	Ct	cyc, pred	Vas020	S
				4	54	2.1	5.8	1	q	Ct	cyc, pred	Vas021	R
11	m	gen	ent, lung, kidney, skin,	1	29	8.9	12.9	0	q	-	-	Vas196	S
			joints, neuro	2	33	12.3	9.5	0	q	-	-	Vas166	S
				3	36	15.9	5.9	0	q	-	-	Vas022	S
				4	42	21.7	0.1	0	q	-	-	Vas191	S
12	m	gen	ent, eyes, kidney, joints	1	77	1.4	0.3	0	a (14)	Ct	aza	Vas023	S
13	m	gen	lung, kidney, skin, joint	1	47	8.9	5.6	0	q	Ct	-	Vas024	R
14	m	gen	ent, kidney, neuro	1	71	8.9	4.3	1	q	Ct	mmf, pred	Vas025	R
				2	71	8.9	4.3	1	q	Ct	mmf, pred	Vas027	R
15*	f	lim	ent	1	32	4.9	19.1	3	a (4)	Ct	cyc, pred	Vas082	S
				2	32	4.9	19.1	3	a (4)	Ct	cyc, pred	Vas083	S
				3	39	11.8	12.2	0	q	Ct	aza, pred	Vas220	R
				4	40	13.3	10.7	0	q	Ct	aza, pred	Vas155	R
				5	41	14.1	9.9	0	q	Ct	aza, pred	Vas160	R
				6	47	20.4	3.6	0	q	Ct	aza, pred	Vas028	R
				7	49	21.8	2.2	0	q	Ct	aza, pred	Vas029	R
				8	51	23.8	0.2	0	q	mupi, mino	pred	Vas186	R
16*	m	lim	ent, lung (trachea)	1	47	3.0	17.8	8	q	Ct	-	Vas131	S
				2	48	3.6	17.3	7	a (4)	-	cyc, pred	Vas132	S
				3	57	13.3	7.6	1	a (4)	Ct	aza, pred	Vas172	R
				4	63	19.0	1.9	0	q	Ct	aza, pred	Vas030	R
				5	65	20.6	0.3	0	q	Ct	aza, pred	Vas031	R
17	f	lim	ent, joints	1	54	3.6	12.6	0	q	-	-	Vas210	S
				2	63	12.7	3.5	0	q	-	-	Vas032	S
				3	67	16.1	0.1	0	q	doxy	-	Vas192	S
18*	m	lim	ent	1	59	3.6	10.9	1	q	Ct	cyc, pred	Vas213	R
				2	60	5.3	9.3	1	q	Ct	aza, pred	Vas153	R
				3	68	13.2	1.4	0	q	Ct	aza, pred	Vas033	R
19	m	gen	ent, lung, kidney, skin	1	29	1.4	12.7	4	q	-	aza	Vas209	S
				2	39	12.0	2.0	1	q	Ct	aza	Vas035	S
20*	m	gen	ent, lung (trachea),	1	37	0.3	20.3	4	q	-	cyc, pred	Vas065	S
			kidney, joints	2	39	2.4	18.2	4	q	-	-	Vas066	S
				3	44	7.8	12.9	4	q	Ct	-	Vas195	R
				4	46	9.8	10.8	4	q	Ct	-	Vas147	R
				5	55	18.6	2.0	1	q	Ct	aza, pred	Vas036	R
				6	57	20.4	0.2	0	q	Ct	pred	Vas189	R
21*	f	gen	ent, kidney, joints,	1	41	3.8	2.0	0	q	Ct	cyc, pred	Vas037	R
			neuro	2	41	4.0	1.8	0	q	Ct	aza, pred	Vas038	R
				3	41	4.3	1.5	0	q	Ct	aza	Vas039	R
				4	42	5.4	0.4	0	q	-	aza	Vas185	S
22*	f	gen	ent, eyes, lung, kidney,	1	54	0.3	17.8	3	q	Ct	cyc, pred	Vas086	S
			skin, joints	2	63	8.5	9.6	0	q	Ct	cyc, pred	Vas154	R
				3	72	17.7	0.4	0	q	Ct	pred	Vas040	R
23*	f	gen	ent, lung, kidney	1	28	3.9	12.6	1	q	Ct	aza	Vas211	R
				2	30	5.6	10.9	1	q	Ct	aza	Vas145	R
				3	40	15.1	1.5	0	q	Ct	-	Vas041	R

				4	40	16.0	0.5	0	q	Ct	-	Vas184	R
24	f	gen	ent, lung, kidney, joints,	1	38	1.9	12.7	1	q	Ct	aza	Vas205	R
			neuro	2	40	3.8	10.8	1	q	Ct	-	Vas150	R
				3	49	12.9	1.8	0	q	Ct	aza, pred	Vas042	R
				4	50	13.7	0.9	0	q	Ct	aza, pred	Vas043	R
				5	50	13.9	0.7	0	q	Ct	aza, pred	Vas180	R
25	m	lim	ent	1	66	10.8	0.9	0	q	Ct	mmf, pred	Vas044	R
				2	66	11.2	0.6	0	q	Ct	mmf, pred	Vas181	R
26*	f	lim	ent	1	45	0.0	12.8	3	a (4)	Ct	-	Vas202	R
				2	47	2.0	10.8	3	q	Ct	mtx	Vas148	R
				3	48	3.0	9.9	3	q	Ct	-	Vas161	R
				4	57	11.9	0.9	0	a (3)	Ct	-	Vas045	R
				5	58	12.6	0.2	0	q	Ct	aza, pred	Vas188	R
27*	f	lim	ent, lung (trachea)	1	33	0.1	9.2	3	q	-	cyc, pred	Vas170	S
				2	41	8.4	0.9	0	q	Ct	cyc	Vas046	S
				3	42	8.8	0.6	0	q	Ct	-	Vas182	S
28*	m	gen	ent, kidney, joints	1	67	13.1	0.9	0	q	Ct	aza, pred	Vas047	R
29*	f	lim	ent, joints	1	43	11.3	0.9	0	q	Ct	-	Vas048	R
30	m	gen	ent, lung, kidney	1	53	8.8	0.9	0	q	-	-	Vas049	S
31	f	gen	kidney, joints	1	65	5.9	19.5	3	q	-	-	Vas050	S
32	m	gen	ent, kidney, lung, joints	1	48	17.5	10.8	3	q	BB	-	Vas054	S
				2	49	18.5	9.7	3	q	-	-	Vas055	S
				3	54	24.0	4.2	2	q	Ct, mupi	-	Vas198	S
33	f	lim	ent, eyes, joints	1	85	7.3	17.1	0	q	-	-	Vas056	S
34	f	lim	ent	1	50	3.7	19.5	0	q	-	-	Vas057	S
				2	51	4.5	18.7	0	q	-	-	Vas058	S
35	f	lim	ent, joints	1	50	4.6	1.3	0	q	Ct, mupi	pred	Vas059	R
				2	51	5.9	0.0	0	q	mino	pred	Vas060	R
36	m	gen	ent, lung, kidney, joints	1	29	3.1	20.7	5	q	-	cyc, pred	Vas061	S
				2	32	6.9	16.9	3	a (4)	Ct	aza	Vas062	S
				3	37	10.9	12.9	2	q	Ct	-	Vas199	R
				4	39	13.0	10.8	2	q	-	-	Vas152	S
37	m	gen	ent, eyes, lung, kidney,	1	59	11.4	6.3	1	q	-	-	Vas063	S
			joints	2	61	13.6	4.2	0	q	-	-	Vas064	S
38	m	gen	eyes, lung, kidney,	1	52	16.7	5.7	5	q	-	cyc, pred	Vas067	S
			joints	2	56	19.9	2.5	3	q	Ct, mupi	cyc, pred	Vas068	S
39	m	gen	ent, kidney	1	42	8.9	19.3	3	q	Ct	cyc, pred	Vas069	S
				2	45	12.2	16.0	2	a (8)	Ct	cyc, pred	Vas070	R
				3	49	16.0	12.2	2	q	Ct	aza	Vas217	S
				4	51	18.7	9.5	1	q	Ct	aza	Vas168	S
40	m	lim	ent, lung	1	75	5.2	11.6	0	q	-	-	Vas071	S
				2	76	5.7	11.0	0	q	-	-	Vas072	S
				3	78	7.8	8.9	0	q	-	-	Vas073	S
				4	82	11.9	4.8	0	q	-	-	Vas208	S
				5	85	15.1	1.6	0	q	-	-	Vas167	S
41*	f	lim	ent, joints	1	37	0.8	17.6	5	q	Ct	-	Vas074	S
				2	46	9.1	9.3	2	q	Ct	pred	Vas171	S
42	f	gen	ent, kidney, joints, skin,	1	21	0.9	22.2	7	a (2)	Ct	pred	Vas075	S
			bowel	2	22	1.6	21.5	6	q	Ct	cyc, pred	Vas076	S
				3	24	4.1	19.0	5	q	Ct	-	Vas077	S
				4	31	10.5	12.6	5	q	-	-	Vas212	S
				5	33	13.3	9.8	4	q	-	-	Vas162	S
43*	m	lim	penis, brains	1	56	0.5	11.4	1	q	-	cyc, pred	Vas078	S
				2	56	1.0	11.0	1	q	-	cyc, pred	Vas079	S
44	m	gen	kidney, joints	1	51	4.2	17.0	5	q	-	-	Vas080	S
				2	52	4.9	16.3	5	q	-	-	Vas081	R
				3	56	9.0	12.1	2	q	Ct	-	Vas221	R
45	f	gen	ent, lung, kidney, joints	1	64	19.9	5.6	0	q	-	pred	Vas084	S
				2	66	22.0	3.5	0	q	-	pred	Vas085	S
46	f	gen	ent, kidney, skin	1	70	13.7	4.0	3	q	-	-	Vas087	S
				2	71	13.9	3.8	3	q	doxy	-	Vas088	S
				3	71	14.3	3.4	2	a (6)	doxy	pred	Vas089	S
47	m	gen	ent, lung, kidney	1	71	0.2	10.6	0	q	mupi	cyc, pred	Vas090	S
48	f	gen	ent, lung, kidney	1	69	11.0	7.2	0	q	Ct	pred	Vas091	R
				2	69	11.0	7.2	0	q	Ct	pred	Vas092	R
				3	69	11.0	7.2	0	q	Ct	pred	Vas093	R
				4	70	12.0	6.3	0	q	Ct	pred	Vas094	R
				5	75	17.6	0.6	0	q	Ct	pred	Vas203	S
49	m	gen	ent, lung, kidney	1	66	15.6	19.0	1	q	-	cyc, pred	Vas095	S
				2	67	16.9	17.8	1	q	Ct	pred	Vas096	S
				3	72	21.8	12.9	1	q	-	-	Vas194	S
50	m	lim	ent, eyes, lung, joints	1	50	7.6	11.6	3	q	Ct	pred	Vas097	R
				2	53	10.0	9.2	2	q	Ct	cyc	Vas098	R
				3	59	16.4	2.7	0	q	Ct	cyc, pred	Vas157	R
				4	60	17.2	2.0	0	q	Ct	aza, pred	Vas159	R
51	f	lim	ent, lung	1	49	0.1	17.3	2	a (2)	-	cyc, pred	Vas099	S
52	f	lim	ent, lung (trachea)	1	75	8.7	3.4	0	q	Ct	-	Vas100	S
53	f	lim	ent, joints, skin	1	67	7.4	12.5	0	q	-	-	Vas101	S
				2	68	8.0	11.8	0	q	-	-	Vas102	S
				3	73	13.7	6.2	0	q	-	-	Vas204	S
54*	m	lim	ent, lung, skin	1	72	4.4	14.2	1	a (5)	-	cyc, pred	Vas103	S
				2	73	4.5	14.1	1	q	-	cyc, pred	Vas104	S
				3	74	5.9	12.7	0	q	-	cyc, pred	Vas105	S
				4	81	12.6	6.1	0	q	Ct	-	Vas201	R
				5	83	14.7	3.9	0	q	Ct	-	Vas156	R
55	m	gen	ent, kidney	1	83	10.9	5.9	0	q	doxy	-	Vas106	S
				2	86	13.6	3.2	0	q	-	-	Vas107	S
56	f	lim	ent, eyes, joints	1	51	5.7	18.9	9	q	-	cyc, pred	Vas110	S
				2	53	8.3	16.3	7	a (3)	Ct	cyc, pred	Vas111	S

				3	57	11.9	12.7	6	a (4)	Ct	aza, pred	Vas206	S
57	m	lim	lung, eyes	1	36	1.3	21.5	9	q	-	cyc, pred	Vas112	S
				2	36	1.7	21.2	9	q	-	cyc, pred	Vas113	S
				3	38	3.5	19.3	7	a (9)	-	cyc, pred	Vas114	S
				4	57	22.6	0.2	0	q		mmf, pred	Vas187	S
58*	m	gen	ent, eyes, lung, kidney, joints, neuro	1	67	4.1	6.9	1	q	amoxy	-	Vas116	S
59	m	lim	ent, lung	1	49	9.1	0.2	0	q	Ct	-	Vas117	S
				2	49	9.3	0.0	0	q	Ct	-	Vas118	S
60	m	gen	ent, joints, kidney	1	64	7.6	9.4	3	q	Ct	aza, pred	Vas119	R
				2	65	8.4	8.5	2	q	Ct, mupi	aza, pred	Vas120	R
				3	68	11.9	5.1	1	q	Ct, mupi	aza	Vas121	R
				4	73	16.3	0.7	0	q	Ct, mupi	mmf, pred	Vas207	R
61	f	gen	ent, eyes, lung, kidney, joints	1	54	17.5	17.5	0	q	-	-	Vas122	S
				2	57	20.7	14.2	0	q	-	-	Vas123	S
				3	60	24.0	11.0	0	q	-	-	Vas197	S
62	f	lim	ent, lung, joints, neuro	1	52	4.1	14.5	4	q	-	pred	Vas124	S
				2	52	4.3	14.3	4	q	-	pred	Vas125	S
				3	60	12.7	5.9	1	q	-	mmf, pred	Vas219	R
63	f	gen	ent, eyes, kidney, joints	1	60	7.6	11.9	0	q	-	-	Vas126	S
				2	66	14.1	5.4	0	q	-	-	Vas214	S
				3	69	16.8	2.7	0	q	-	-	Vas163	S
64	f	gen	lung, kidney, bowel	1	28	5.0	19.4	0	q	-	-	Vas127	S
				2	29	6.5	17.8	0	q	Ct	-	Vas128	S
				3	34	11.5	12.9	0	q	-	-	Vas193	S
				4	38	15.0	9.4	0	q	Ct	-	Vas169	S
65	m	gen	ent, lung, kidney, joints, neuro	1	85	4.1	2.5	0	q	-	cyc	Vas129	R
				2	86	5.2	1.3	0	q	-	-	Vas130	S
66	m	gen	ent, kidney, skin, bowel, neuro	1	28	3.9	19.8	1	q	-	aza, pred	Vas133	S
				2	29	5.3	18.3	0	q	-	aza, pred	Vas134	S
67*	f	gen	eyes, lung, kidney, joints	1	50	4.9	10.7	0	q	Ct	-	Vas135	S
				2	54	9.2	6.3	0	q	-	-	Vas136	S
68	f	gen	ent, lung, kidney, joints, neuro	1	61	1.0	17.8	0	q	-	cyc, pred	Vas137	S
				2	63	2.8	15.9	0	q	-	-	Vas138	S
				3	68	7.7	11.1	0	q	-	-	Vas215	S
				4	71	11.1	7.6	0	q	-	-	Vas173	S
69	f	gen	eyes, kidney, skin, joints, neuro	1	38	1.2	19.5	2	q	-	cyc, pred	Vas139	S
				2	40	3.2	17.5	1	q	-	cyc	Vas140	S
70	f	gen	ent, eyes, kidney	1	64	1.9	4.7	0	q	-	-	Vas141	S
				2	67	4.4	2.2	0	q	-	-	Vas142	S
71*	f	gen	ent, eyes, lung, kidney, joints, neuro, skin, muscles	1	28	0.2	18.5	13	q	-	cyc, pred	Vas143	S
				2	30	2.0	16.7	13	q	-	aza	Vas144	S
				3	34	6.3	12.4	8	q	-	aza, pred	Vas216	S
				4	37	9.0	9.7	6	q	Ct	pred	Vas164	S
				5	46	18.1	0.6	1	q	Ct	mmf, pred	Vas183	R

Abbreviations: pat. = patient; *= selected for Luminex analysis; gen = generalized disease (including kidney involvement); lim = limited disease (upper and/or lower airway involvement not accompanied by glomerulonephritis); a = active (BVAS score); q = quiescent; Ct = co-trimoxazole; mupi = mupirocin; mino = minocyclin; doxy = doxycyclin; amoxy = amoxicillin; mmf = mycophenolatemophetil; pred = prednisolone; aza = azathioprine; cyc = cyclophosphamide; mtx = methotrexate; R = resistance; S = sensitive.

Supplementary Table 3. Characteristics of the 210 *S. aureus* isolates from GPA patients displayed in the order of the MLVF dendrogram and the 18 *S. aureus* isolates from the HC carriers. *spa*-typing was performed as previously described [1]. Briefly, the protein A (*spa*) gene of *S. aureus* was PCR amplified and sequenced. Based on the detected variable number tandem repeats (VNTRs) and their composition, a specific *spa*-type was assigned using the Ridom StaphType software (version 2.2.1; Ridom GmbH, Würzburg, Germany) and the Ridom *Spa* Server (www.spaserver.ridom.de) [2]. To determine the clonal relatedness of the *S. aureus* population, the based upon repeat patterns (BURP) algorithm was applied (Ridom StaphType software 2.2.1) [2]. *Spa*-types with more than five repeats were clustered into different groups, the calculated cost between members of a group being ≤ 4 [2]. The MLVF experiment, including DNA isolation, multiplex PCR, separation of PCR products on the Bioanalyzer 2100 (Agilent Technologies), and data analyses with GelCompar II (Applied Maths, Sint-Martens-Latem, Belgium) were performed as recently described [3,4]. The position tolerance and optimization were set to 0.5% and 0.5%, respectively, and the dice formula was used to calculate the pairwise similarity coefficient. With the selected position tolerance, all Bioanalyzer runs for the control isolate M2 were identical. After visual inspection of the MLVF dendrogram, six different cut-off values (89%, 81%, 70%, 66%, 64%, 60% and 50%) were chosen for testing the concordance between MLVF and *spa*-typing.

Sample	Patient Number	Isolate Number	Isolation Year	*spa*-type	Repeat Succession	MLVF pattern	MLVF cluster (66%)	*spa*-CC	Co-trimoxazole phenotype	*tst*-1
Vas124	62	1	1992	t021	15-12-16-02-16-02-25-17-24	1	C1	spa-CC 012	S	neg
Vas125	62	2	1992	t021	15-12-16-02-16-02-25-17-24	1	C1	spa-CC 012	S	neg
Vas210	17	1	2000	t435	14-44-13-17-17-17-23-18-17	2	C1	singleton 8	S	neg
Vas189	20	6	2012	t072	26-23-23-17-12-17-16	3	C2	spa-CC 062	R	neg
Vas036	20	5	2010	t072	26-23-23-17-12-17-16	3	C2	spa-CC 062	R	neg
Vas214	63	2	2000	t1306	15-12-16-02-16-02-16-02-25-17-24-24	4	C3	spa-CC 012	S	pos
Vas055	32	2	1994	t238	15-21-12-16-02-16-02-25-17-24-24	5	C3	spa-CC 012	S	pos
Vas054	32	1	1993	t012	15-12-16-02-16-02-25-17-24-24	6	C3	spa-CC 012	S	pos
Vas101	53	1	1993	t012	15-12-16-02-16-02-25-17-24-24	7	C3	spa-CC 012	S	pos
Vas102	53	2	1994	t012	15-12-16-02-16-02-25-17-24-24	7	C3	spa-CC 012	S	pos
Vas135	67	1	1991	t012	15-12-16-02-16-02-25-17-24-24	7	C3	spa-CC 012	S	pos
Vas056	33	1	1995	t012	15-12-16-02-16-02-25-17-24-24	7	C3	spa-CC 012	S	pos
Vas074	41	1	1995	t012	15-12-16-02-16-02-25-17-24-24	7	C3	spa-CC 012	S	pos
Vas136	67	2	1995	t021	15-12-16-02-16-02-25-17-24	8	C3	spa-CC 012	S	pos
Vas075	42	1	1990	t012	15-12-16-02-16-02-25-17-24-24	9	C3	spa-CC 012	S	pos
Vas076	42	2	1991	t012	15-12-16-02-16-02-25-17-24-24	9	C3	spa-CC 012	S	pos
Vas212	42	4	2000	t012	15-12-16-02-16-02-25-17-24-24	10	C3	spa-CC 012	S	neg
Vas095	49	1	1993	t685	15-12-16-02-16-02-24-24	11	C4	spa-CC 012	S	pos
Vas096	49	2	1995	t685	15-12-16-02-16-02-24-24	11	C4	spa-CC 012	S	pos
Vas063	37	1	1993	t021	15-12-16-02-16-02-25-17-24	12	C5	spa-CC 012	S	pos
Vas064	37	2	1995	t021	15-12-16-02-16-02-25-17-24	12	C5	spa-CC 012	S	pos
Vas157	50	3	2002	t021	15-12-16-02-16-02-25-17-24	13	C5	spa-CC 012	R	neg
Vas159	50	4	2003	t021	15-12-16-02-16-02-25-17-24	13	C5	spa-CC 012	R	neg
Vas062	36	2	1996	t021	15-12-16-02-16-02-25-17-24	14	C5	spa-CC 012	S	pos
Vas143	71	1	1994	t021	15-12-16-02-16-02-25-17-24	15	C5	spa-CC 012	S	neg
Vas047	28	1	2012	t021	15-12-16-02-16-02-25-17-24	16	C5	spa-CC 012	R	neg
Vas192	17	3	2012	t089	04-33-31-12-16-34-16-12-33-34	17	C6	singleton 4	S	pos
Vas032	17	2	2009	t089	04-33-31-12-16-34-16-12-33-34	17	C6	singleton 4	S	pos
Vas167	40	5	2003	t1077	14-44-12-17-23-18-17	18	C6	singleton 9	S	neg
Vas208	40	4	2000	t12042	14-44-12-17-17-23-24	18	C6	singleton 15	S	neg
Vas071	40	1	1993	t1077	14-44-12-17-23-18-17	18	C6	singleton 9	S	neg
Vas072	40	2	1994	t1077	14-44-12-17-23-18-17	18	C6	singleton 9	S	neg
Vas073	40	3	1996	t1077	14-44-12-17-23-18-17	18	C6	singleton 9	S	neg
Vas087	46	1	1993	t318	15-12-16-16-02-16-02-25-17-24	19	C7	spa-CC 012	S	pos
Vas088	46	2	1993	t318	15-12-16-16-02-16-02-25-17-24	19	C7	spa-CC 012	S	pos
Vas089	46	3	1993	t318	15-12-16-16-02-16-02-25-17-24	19	C7	spa-CC 012	S	pos
Vas127	64	1	1993	t012	15-12-16-02-16-02-25-17-24-24	20	C7	spa-CC 012	S	pos
Vas170	27	1	2003	t018	15-12-16-02-16-02-25-17-24-24-24	21	C7	spa-CC 012	S	pos
Vas049	30	1	2012	t015	08-16-02-16-34-13-17-34-16-34	22	C8	singleton 1	S	neg
Vas194	49	3	2000	t196	04-34-32-17-23-24	23	C9	singleton 7	S	neg
Vas018	10	1	2006	t539	26-23-17-34-17-12-17-16	24	C10	spa-CC 062	S	neg
Vas019	10	2	2006	t539	26-23-17-34-17-12-17-16	24	C10	spa-CC 062	S	neg
Vas020	10	3	2006	t539	26-23-17-34-17-12-17-16	24	C10	spa-CC 062	S	neg
Vas021	10	4	2006	t539	26-23-17-34-17-12-17-16	24	C10	spa-CC 062	R	neg
Vas133	66	1	1993	t164	07-06-17-21-34-34-22-34	25	C10	singleton 6	S	neg
Vas134	66	2	1994	t164	07-06-17-21-34-34-22-34	25	C10	singleton 6	S	neg
Vas009	6	1	2006	t3092	07-23-12-21-12-17-20-17-12-17	26	C10	singleton 13	S	neg
Vas130	65	2	1996	t062	26-23-17-12-17-16	27	C11	spa-CC 062	S	neg
Vas050	31	1	1993	t062	26-23-17-12-17-16	27	C11	spa-CC 062	S	neg
Vas187	57	4	2012	t002	26-23-17-34-17-20-17-12-17-16	28	C11	spa-CC 062	S	neg
Vas110	56	1	1994	t002	26-23-17-34-17-20-17-12-17-16	29	C11	spa-CC 062	S	neg
Vas086	22	1	1993	t002	26-23-17-34-17-20-17-12-17-16	29	C11	spa-CC 062	S	neg
Vas010	7	2	2006	t224	07-23-12-21-17-34-33-34	30	C11	no founder	R	neg
Vas158	7	1	2002	t002	26-23-17-34-17-20-17-12-17-16	30	C11	spa-CC 062	S	neg
Vas106	55	1	1993	NT		31	C12	NT	S	neg
Vas163	63	3	2003	t2050	07-23-21-02-34	32	C12	singleton 12	S	neg
Vas152	36	4	2002	t034	08-16-02-25-02-25-34-24-25	33	C13	singleton 2	S	neg
Vas023	12	1	2007	t1265	26-23-17-34-17-20-17-12-12-12-16	34	C13	spa-CC 062	S	neg
Vas084	45	1	1993	t246	04-17-23-24-20-17-25	35	C14	no founder	S	neg
Vas085	45	2	1995	t246	04-17-23-24-20-17-25	35	C14	no founder	S	neg
Vas078	43	1	1994	t177	26-23-21-16-34-33-13	36	C14	no founder	S	neg
Vas185	21	4	2012	t4584	26-23-13-23-31-05-17-25-17-25-23	37	C14	singleton 14	S	neg
Vas122	61	1	1993	t026	08-16-34	38	C15	excluded	S	neg
Vas123	61	2	1996	t026	08-16-34	38	C15	excluded	S	neg
Vas111	56	2	1996	t026	08-16-34	39	C15	excluded	S	neg
Vas116	58	1	1996	t026	08-16-34	39	C15	excluded	S	neg
Vas144	71	2	1996	t026	08-16-34	40	C15	excluded	S	neg

Vas197	61	3	2000	t026	08-16-34	41	C15	excluded	S	neg
Vas061	36	1	1992	t026	08-16-34	42	C15	excluded	S	neg
Vas001	1	2	2006	t091	07-23-21-17-34-12-23-02-12-23	43	C16	singleton 5	S	neg
Vas155	15	4	2002	t091	07-23-21-17-34-12-23-02-12-23	43	C16	singleton 5	R	neg
Vas160	15	5	2003	t091	07-23-21-17-34-12-23-02-12-23	43	C16	singleton 5	R	neg
Vas172	16	3	2003	t091	07-23-21-17-34-12-23-02-12-23	43	C16	singleton 5	R	neg
Vas186	15	8	2012	t091	07-23-21-17-34-12-23-02-12-23	43	C16	singleton 5	R	neg
Vas190	1	3	2012	t091	07-23-21-17-34-12-23-02-12-23	43	C16	singleton 5	R	neg
Vas198	32	3	2000	t091	07-23-21-17-34-12-23-02-12-23	43	C16	singleton 5	R	neg
Vas204	53	3	2000	t091	07-23-21-17-34-12-23-02-12-23	43	C16	singleton 5	R	neg
Vas220	15	3	2000	t091	07-23-21-17-34-12-23-02-12-23	43	C16	singleton 5	R	neg
Vas025	14	1	2008	t091	07-23-21-17-34-12-23-02-12-23	43	C16	singleton 5	R	neg
Vas027	14	1	2008	t091	07-23-21-17-34-12-23-02-12-23	43	C16	singleton 5	R	neg
Vas028	15	6	2009	t091	07-23-21-17-34-12-23-02-12-23	43	C16	singleton 5	R	neg
Vas029	15	7	2010	t091	07-23-21-17-34-12-23-02-12-23	43	C16	singleton 5	R	neg
Vas030	16	4	2009	t091	07-23-21-17-34-12-23-02-12-23	43	C16	singleton 5	R	neg
Vas031	16	5	2010	t091	07-23-21-17-34-12-23-02-12-23	43	C16	singleton 5	R	neg
Vas037	21	1	2010	t091	07-23-21-17-34-12-23-02-12-23	43	C16	singleton 5	S	neg
Vas038	21	2	2011	t091	07-23-21-17-34-12-23-02-12-23	43	C16	singleton 5	S	neg
Vas039	21	3	2011	t091	07-23-21-17-34-12-23-02-12-23	43	C16	singleton 5	R	neg
Vas011	8	2	2006	t064	11-19-12-05-17-34-24-34-22-25	44	C17	spa-CC 064	R	neg
Vas145	23	2	2002	t064	11-19-12-05-17-34-24-34-22-25	44	C17	spa-CC 064	R	neg
Vas147	20	4	2002	t064	11-19-12-05-17-34-24-34-22-25	44	C17	spa-CC 064	R	neg
Vas149	5	3	2002	t064	11-19-12-05-17-34-24-34-22-25	44	C17	spa-CC 064	R	neg
Vas150	24	2	2002	t064	11-19-12-05-17-34-24-34-22-25	44	C17	spa-CC 064	R	neg
Vas153	18	2	2002	t2658	11-19-36-05-17-34-24-34-22-25	44	C17	spa-CC 064	R	neg
Vas156	54	5	2002	t064	11-19-12-05-17-34-24-34-22-25	44	C17	spa-CC 064	R	neg
Vas180	24	5	2012	t064	11-19-12-05-17-34-24-34-22-25	44	C17	spa-CC 064	R	neg
Vas181	25	2	2012	t064	11-19-12-05-17-34-24-34-22-25	44	C17	spa-CC 064	R	neg
Vas195	20	3	2000	t064	11-19-12-05-17-34-24-34-22-25	44	C17	spa-CC 064	R	neg
Vas199	36	3	2000	t064	11-19-12-05-17-34-24-34-22-25	44	C17	spa-CC 064	R	neg
Vas201	54	4	2000	t064	11-19-12-05-17-34-24-34-22-25	44	C17	spa-CC 064	R	neg
Vas205	24	1	2000	t064	11-19-12-05-17-34-24-34-22-25	44	C17	spa-CC 064	R	neg
Vas207	60	4	2000	t064	11-19-12-05-17-34-24-34-22-25	44	C17	spa-CC 064	R	neg
Vas211	23	1	2000	t064	11-19-12-05-17-34-24-34-22-25	44	C17	spa-CC 064	R	neg
Vas213	18	1	2000	t2658	11-19-36-05-17-34-24-34-22-25	44	C17	spa-CC 064	R	neg
Vas219	62	3	2000	t064	11-19-12-05-17-34-24-34-22-25	44	C17	spa-CC 064	R	neg
Vas222	8	1	2000	t064	11-19-12-05-17-34-24-34-22-25	44	C17	spa-CC 064	R	neg
Vas024	13	1	2007	t064	11-19-12-05-17-34-24-34-22-25	44	C17	spa-CC 064	R	neg
Vas033	18	3	2010	t2658	11-19-36-05-17-34-24-34-22-25	44	C17	spa-CC 064	R	neg
Vas041	23	3	2011	t12249	11-12-05-17-34-24-34-17-22-25	44	C17	spa-CC 064	R	neg
Vas042	24	3	2011	t064	11-19-12-05-17-34-24-34-22-25	44	C17	spa-CC 064	R	neg
Vas043	24	4	2011	t064	11-19-12-05-17-34-24-34-22-25	44	C17	spa-CC 064	R	neg
Vas048	29	1	2012	t064	11-19-12-05-17-34-24-34-22-25	44	C17	spa-CC 064	R	neg
Vas007	4	1	2006	t064	11-19-12-05-17-34-24-34-22-25	44	C17	spa-CC 064	R	neg
Vas070	39	2	1996	t064	11-19-12-05-17-34-24-34-22-25	44	C17	spa-CC 064	R	neg
Vas081	44	2	1996	t064	11-19-12-05-17-34-24-34-22-25	44	C17	spa-CC 064	R	neg
Vas221	44	3	2000	t064	11-19-12-05-17-34-24-34-22-25	45	C17	spa-CC 064	R	neg
Vas044	25	1	2011	t064	11-19-12-05-17-34-24-34-22-25	46	C17	spa-CC 064	R	neg
Vas004	3	1	2006	t064	11-19-12-05-17-34-24-34-22-25	47	C17	spa-CC 064	R	neg
Vas040	22	4	2011	t064	11-19-12-05-17-34-24-34-22-25	47	C17	spa-CC 064	R	neg
Vas005	3	2	2006	t064	11-19-12-05-17-34-24-34-22-25	47	C17	spa-CC 064	R	neg
Vas006	3	3	2006	t064	11-19-12-05-17-34-24-34-22-25	47	C17	spa-CC 064	R	neg
Vas161	26	3	2003	t064	11-19-12-05-17-34-24-34-22-25	48	C17	spa-CC 064	R	neg
Vas188	26	5	2012	t064	11-19-12-05-17-34-24-34-22-25	48	C17	spa-CC 064	R	neg
Vas202	26	1	2000	t064	11-19-12-05-17-34-24-34-22-25	48	C17	spa-CC 064	R	neg
Vas148	26	2	2002	t064	11-19-12-05-17-34-24-34-22-25	49	C17	spa-CC 064	R	neg
Vas196	11	1	2000	t1246	04-21-12-41-20-17-12-12-12-41	50	C17	singleton 10	S	neg
Vas151	2	1	2002	t064	11-19-12-05-17-34-24-34-22-25	51	C17	spa-CC 064	R	neg
Vas174	2	2	2003	t064	11-19-12-05-17-34-24-34-22-25	51	C17	spa-CC 064	R	neg
Vas002	2	3	2006	t064	11-19-12-05-17-34-24-34-22-25	51	C17	spa-CC 064	R	neg
Vas003	2	4	2012	t091	07-23-21-17-34-12-23-02-12-23	51	C17	singleton 5	R	neg
Vas184	23	4	2012	t12249	11-12-05-17-34-24-34-17-22-25	52	C17	spa-CC 064	R	neg
Vas012	8	3	2006	t064	11-19-12-05-17-34-24-34-22-25	53	C17	spa-CC 064	R	neg
Vas077	42	3	1993	t842	11-34-22-25	53	C17	excluded	S	neg
Vas209	19	1	2000	t211	19-12-12-21-17-34-24-34-22-25	54	C17	spa-CC 064	S	neg
Vas169	64	4	2003	t12246	04-12-21-12-17-20-17-12-12-17	55	C17	singleton 16	S	neg
Vas193	64	3	2000	t12246	04-12-21-12-17-20-17-12-12-17	55	C17	singleton 16	S	neg
Vas015	9	2	2006	t064	11-19-12-05-17-34-24-34-22-25	56	C17	spa-CC 064	R	neg
Vas016	9	3	2006	t064	11-19-12-05-17-34-24-34-22-25	56	C17	spa-CC 064	R	neg
Vas017	9	4	2006	t064	11-19-12-05-17-34-24-34-22-25	56	C17	spa-CC 064	R	neg
Vas175	9	1	2005	t064	11-19-12-05-17-34-24-34-22-25	56	C17	spa-CC 064	R	neg
Vas035	19	2	2010	t3092	07-23-12-21-12-17-20-17-12-17	57	C17	singleton 13	S	neg
Vas008	5	4	2006	t064	11-19-12-05-17-34-24-34-22-25	57	C17	spa-CC 064	S	neg
Vas100	52	1	1994	t12248	08-16-02-16-13-13-34-34-16-34	58	C17	singleton 17	S	neg
Vas117	59	1	1991	t015	08-16-02-16-34-13-17-34-16-34	59	C17	singleton 1	S	neg
Vas118	59	2	1991	t015	08-16-02-16-34-13-17-34-16-34	59	C17	singleton 1	S	neg
Vas013	8	4	2010	t064	11-19-12-05-17-34-24-34-22-25	60	C17	spa-CC 064	R	neg
Vas014	8	5	2011	t064	11-19-12-05-17-34-24-34-22-25	60	C17	spa-CC 064	R	neg
Vas079	43	2	1994	t064	11-19-12-05-17-34-24-34-22-25	60	C17	spa-CC 064	S	neg
Vas178	8	6	2012	t064	11-19-12-05-17-34-24-34-22-25	61	C17	spa-CC 064	R	neg
Vas166	11	2	2003	t1246	04-21-12-41-20-17-12-12-12-41	62	C18	singleton 10	S	neg
Vas022	11	3	2007	t1246	04-21-12-41-20-17-12-12-12-41	62	C18	singleton 10	S	neg
Vas191	11	4	2012	t1246	04-21-12-41-20-17-12-12-12-41	63	C18	singleton 10	S	neg
Vas182	27	3	2012	t1508	15-16-34-33-13	64	C19	singleton 11	S	neg
Vas046	27	2	2011	t1508	15-16-34-33-13	64	C19	singleton 11	S	neg
Vas183	71	5	2012	t1361	08-23-02-12-23	65	C19	no founder	R	neg
Vas137	68	1	1993	t127	07-23-21-16-34-33-13	66	C20	no founder	S	neg
Vas138	68	2	1995	t127	07-23-21-16-34-33-13	66	C20	no founder	S	neg

Vas215	68	3	2000	t127	07-23-21-16-34-33-13	67	C20	no founder	S	neg
Vas045	26	4	2011	t451	11-12-05-17-34-24-34-22-25	68	C20	spa-CC 064	R	neg
Vas173	68	4	2003	t1727	07-23-12-34-34-34-34-12-12-23-02-12-23	69	C21	spa-CC 084	S	neg
Vas065	20	1	1992	t359	07-23-12-21-17-34-34-33-34	70	C21	no founder	S	neg
Vas052	5	1	1996	t180	09-02-16-34-34-34-17-34-16-34	71	C21	spa-CC 330/180	S	neg
Vas053	5	2	1996	t12247	09-34-16-34-34-34-17-34-16-34	71	C21	spa-CC 330/180	S	neg
Vas131	16	1	1993	t330	09-02-16-34-34-17-34-16-34	72	C21	spa-CC 330/180	S	neg
Vas132	16	2	1993	t065	09-02-16-34-13-17-34-16-34	72	C21	spa-CC 330/180	S	neg
Vas162	42	5	2003	t065	09-02-16-34-13-17-34-16-34	73	C22	spa-CC 330/180	S	neg
Vas216	71	3	2000	t026	08-16-34	74	C22	excluded	S	neg
Vas203	48	5	2000	t043	09-02-16-13-13-17-13-16-34	75	C23	spa-CC 330/180	S	neg
Vas059	35	1	1995	t8351	04-17-23-24-20-24-20-17-25	76	C24	no founder	R	neg
Vas060	35	2	1996	t8351	04-17-23-24-20-24-20-17-25	76	C24	no founder	R	neg
Vas168	39	4	2003	t056	04-20-12-17-20-17-12-17-17	77	C25	singleton 3	S	neg
Vas217	39	3	2000	t056	04-20-12-17-20-17-12-17-17	77	C25	singleton 3	S	neg
Vas069	39	1	1993	t056	04-20-12-17-20-17-12-17-17	77	C25	singleton 3	S	neg
Vas097	50	1	1993	t120	07-23-12-12-34-34-12-12-23-02-12-23	78	C26	spa-CC 084	R	neg
Vas098	50	2	1995	t120	07-23-12-12-34-34-12-12-23-02-12-23	78	C26	spa-CC 084	R	neg
Vas112	57	1	1991	t084	07-23-12-34-34-12-12-23-02-12-23	79	C26	spa-CC 084	S	neg
Vas129	65	1	1995	t084	07-23-12-34-34-12-12-23-02-12-23	79	C26	spa-CC 084	S	neg
Vas099	51	1	1995	t084	07-23-12-34-34-12-12-23-02-12-23	79	C26	spa-CC 084	R	neg
Vas128	64	2	1995	t084	07-23-12-34-34-12-12-23-02-12-23	80	C26	spa-CC 084	S	neg
Vas066	20	2	1994	t084	07-23-12-34-34-12-12-23-02-12-23	80	C26	spa-CC 084	S	neg
Vas067	38	1	1993	t084	07-23-12-34-34-12-12-23-02-12-23	81	C26	spa-CC 084	S	neg
Vas091	48	1	1993	t084	07-23-12-34-34-12-12-23-02-12-23	82	C26	spa-CC 084	R	neg
Vas171	41	2	2003	t11401	07-23-21-02-12-23	83	C26	no founder	S	neg
Vas057	34	1	1993	t328	07-23-12-34-34-12-12-23-02-12-23-02-12-23	83	C26	spa-CC 084	S	neg
Vas058	34	2	1994	t328	07-23-12-34-34-12-12-23-02-12-23-02-12-23	84	C26	spa-CC 084	S	neg
Vas154	22	3	2002	t346	07-23-12-34-12-12-23-02-12-23	85	C26	spa-CC 084	S	neg
Vas090	47	1	1990	t346	07-23-12-34-12-12-23-02-12-23	85	C26	spa-CC 084	R	neg
Vas082	15	1	1993	t084	07-23-12-34-34-12-12-23-02-12-23	86	C26	spa-CC 084	S	neg
Vas083	15	2	1993	t084	07-23-12-34-34-12-12-23-02-12-23	86	C26	spa-CC 084	S	neg
Vas104	54	2	1992	t084	07-23-12-34-34-12-12-23-02-12-23	87	C26	spa-CC 084	R	neg
Vas105	54	3	1993	t084	07-23-12-34-34-12-12-23-02-12-23	87	C26	spa-CC 084	R	neg
Vas113	57	2	1991	t084	07-23-12-34-34-12-12-23-02-12-23	87	C26	spa-CC 084	R	neg
Vas114	57	3	1993	t084	07-23-12-34-34-12-12-23-02-12-23	87	C26	spa-CC 084	S	neg
Vas126	63	1	1993	t084	07-23-12-34-34-12-12-23-02-12-23	87	C26	spa-CC 084	S	neg
Vas139	69	1	1993	t084	07-23-12-34-34-12-12-23-02-12-23	87	C26	spa-CC 084	S	neg
Vas140	69	2	1995	t084	07-23-12-34-34-12-12-23-02-12-23	87	C26	spa-CC 084	S	neg
Vas141	70	1	1993	t5721	07-34-12-12-23-02-12-23-02-12-23	87	C26	spa-CC 084	S	neg
Vas142	70	2	1995	t5721	07-34-12-12-23-02-12-23-02-12-23	87	C26	spa-CC 084	S	neg
Vas146	1	1	2002	t084	07-23-12-34-34-12-12-23-02-12-23	87	C26	spa-CC 084	S	neg
Vas092	48	2	1993	t084	07-23-12-34-34-12-12-23-02-12-23	87	C26	spa-CC 084	S	neg
Vas093	48	3	1993	t084	07-23-12-34-34-12-12-23-02-12-23	87	C26	spa-CC 084	S	neg
Vas094	48	4	1994	t084	07-23-12-34-34-12-12-23-02-12-23	87	C26	spa-CC 084	S	neg
Vas206	56	3	2000	t084	07-23-12-34-34-12-12-23-02-12-23	88	C26	spa-CC 084	S	neg
Vas107	55	2	1996	t084	07-23-12-34-34-12-12-23-02-12-23	89	C26	spa-CC 084	S	neg
Vas068	38	2	1996	t328	07-23-12-34-34-12-12-23-02-12-23-02-12	90	C26	spa-CC 084	S	neg
Vas103	54	1	1991	NT		91	C27	NT	S	neg
Vas119	60	1	1991	t084	07-23-12-34-34-12-12-23-02-12-23	92	C28	spa-CC 084	R	neg
Vas120	60	2	1992	t084	07-23-12-34-34-12-12-23-02-12-23	92	C28	spa-CC 084	R	neg
Vas121	60	3	1995	t084	07-23-12-34-34-12-12-23-02-12-23	93	C28	spa-CC 084	R	neg
Vas164	71	4	2003	t084	07-23-12-34-34-12-12-23-02-12-23	94	C29	spa-CC 084	S	neg
Vas080	44	1	1995	t085	07-23-12-34-34-12-23-02-12-23	95	C30	spa-CC 084	S	neg
CC29	HC1	1	2007	t122	08-16-02-16-02-25-17-24-24	ND	ND	ND	S	pos
CC31	HC1	2	2010	t122	08-16-02-16-02-25-17-24-24	ND	ND	ND	S	pos
CC32	HC2	3	2007	t012	15-12-16-02-16-02-25-17-24-24	ND	ND	ND	S	pos
CC70	HC2	1	2010	t091	07-23-21-17-34-12-23-02-12-23	ND	ND	ND	S	neg
CC47	HC3	2	2007	t2109	07-83-22-34	ND	ND	ND	S	neg
CC1	HC3	1	2010	t2109	07-83-22-34	ND	ND	ND	S	neg
CC57	HC4	2	2007	t008	11-19-12-21-17-34-24-34-22-25	ND	ND	ND	S	neg
IEC3	HC4	1	2008	t024	11-12-21-17-34-24-34-22-25	ND	ND	ND	S	neg
CC82	HC4	2	2010	t6076	26-23-20-05-17-25-17-25-16-28	ND	ND	ND	S	neg
CC7	HC8	1	2010	t209	07-16-12-23-34	ND	ND	ND	S	neg
CC25	HC9	2	2010	t091	07-23-21-17-34-12-23-02-12-23	ND	ND	ND	S	neg
CC76	HC10	1	2007	t012	15-12-16-02-16-02-25-17-24-24	ND	ND	ND	S	pos
IEC8	HC10	2	2008	t012	15-12-16-02-16-02-25-17-24-24	ND	ND	ND	S	pos
CC26	HC11	1	2007	t002	26-23-17-34-17-20-17-12-17-16	ND	ND	ND	S	pos
IEC10	HC11	2	2008	t359	07-23-12-21-17-34-34-33-34	ND	ND	ND	S	neg
CC38	HC14	1	2007	t021	15-12-16-02-16-02-25-17-24	ND	ND	ND	S	pos
IEC11	HC14	2	2008	t021	15-12-16-02-16-02-25-17-24	ND	ND	ND	S	pos
CC107	HC15	1	2007	t230	08-16-02-16-34	ND	ND	ND	S	neg

Abbreviations: pos = positive, neg = negative, C1 = Cluster 1, *spa*-CC 084 = *spa* clonal complex 084, NT = not typeable, ND = not determined, S = sensitive, R = resistant

References

1. Harmsen, D. *et al.* Typing of methicillin-resistant *Staphylococcus aureus* in a university hospital setting by using novel software for *spa* repeat determination and database management. *J. Clin. Microbiol.* **41,** 5442–5448 (2003).
2. Mellmann, A. *et al.* Based Upon Repeat Pattern (BURP): an algorithm to characterize the long-term evolution of S*taphylococcus aureus* populations based on *spa* polymorphisms. *BMC Microbiol.* **7,** 98 (2007).
3. Glasner, C. *et al.* Rapid and high-resolution distinction of community-acquired and nosocomial *Staphylococcus aureus* isolates with identical pulsed-field gel electrophoresis patterns and *spa* types. *Int. J. Med. Microbiol.* **303,** 70–75 (2013).

4. Sabat, A. J. *et al.* Microfluidic chip-based multiple-locus variable-number tandem repeat fingerprinting (MLVF) with new primer sets for methicillin-resistant *Staphylococcus aureus*. *J. Clin. Microbiol.* **50,** 2255–2262 (2012).

Supplementary Table 4. Information on *Staphylococcus aureus* antigens used in the multiplex *S. aureus* antibody assay. The relative amounts of serum IgGs against 59 *S. aureus* antigens were determined by bead-based Luminex flow cytometry (xMAP®, Luminex Corporation, Austin, Texas, USA) [1]. Independent duplicate Luminex assays were performed on different days, and the median fluorescence intensity (MFI), reflecting quantitative antibody levels, was averaged. To determine non-specific binding, control beads without coupled protein were included and this MFI value was subtracted from the antigen-specific MFI [1,2].

Abbreviation	Explanation	Function
21 superantigens and superantigen -like proteins		
SEA-SEE; SEG-SEJ	Staphylococcal enterotoxins A-E; G-J	Non-specific binding to T-cell receptor and MHC II of antigen presenting cells causing polyclonal T cell stimulation
SEM-SEO; SEQ; SER	Staphylococcal enterotoxins M-O; Q; R	
TSST-1	Toxic shock syndrome toxin 1	
SSL1, 3, 5, 9-11	Staphylococcal superantigen-like proteins 1, 3, 5, 9-11	Stimulation of innate immunity (i.e. complement, Fc receptors, myeloid cells), but not T cells
10 surface proteins		
ClfA, ClfB	Clumping factor A, B	Adhesins, bind to fibrinogen
SdrD, SdrE	Serine-aspartate dipeptide repeat containing protein D, E	Adhesins, role in platelet aggregation and adherence
FnbpA, FnbpB	Fibronectin-binding protein A, B	Adhesins, bind to fibronectin
IsdA, IsdH	Iron-regulated surface determinants A, H	Bind to iron-containing proteins, such as transferrin, haemin, haemoglobulin
SasG	*S. aureus* surface protein G	Adhesin
PrsA	Foldase protein	Folding of secreted proteins
26 secreted proteins		
ETA, ETB	Exfoliative toxins A and B	Causative agents of staphylococcal scalded skin syndrome; serine proteases which selectively recognize and cleave desmosomal proteins in the skin
CHIPS	Chemotaxis inhibitory protein of *S. aureus*	Inhibiton of neutrophil and monocyte chemotaxis through binding to C5a receptor and formylpeptide receptor
SCIN	Staphylococcal complement inhibitor	Complement inhibitor
Luk D, E, F, S	Leukocidins D, E, F, S	Membrane-damaging toxins causing disruption and lysis of cells
HlgB, α-toxin, Hlb	Gamma-, alpha-, and beta-hemolysin	
IsaA	Immunodominant staph. antigen A	Lytic transglycolase that cleaves peptidoglycan
LytM	Glycylglycine endopeptidase	Degrades the peptidoglycan cell wall
NUC	Nuclease	Catalyses the hydrolysis of DNA and RNA
EsxA, EsxB	ESAT-6 secretion system extracellular A, B	Transport chaperone/adaptor proteins
Lipase	Lipase	Lipid digestion
FlipR, FlipR-L	Formyl peptide receptor-like inhibitory proteins	Inhibitor of neutrophil activation
EAP, EapH1, EapH2	Extracellular adherence proteins	Activator of extracellular proteases
Pro-Atl	SA0905 gene; N-terminus of bifunctional autolysin	Propeptide of Atl
Atl-2	SA0905 gene; C-terminus of bifunctional autolysin	Glucosaminidase
Aly	SA2437 gene; autolysin precursor	
Efb	Extracellular fibrinogen-binding protein	Adhesin, binds fibrinogen and C3
2 unknown/hypothetical proteins		

SA0104	SA0104 gene; norG protein	HTH-type transcriptional regulator
SA2097	SA2097 gene; hypothetical protein	?

References

1. Verkaik, N. J. *et al.* Anti-staphylococcal humoral immune response in persistent nasal carriers and noncarriers of *Staphylococcus aureus*. *J Infect Dis* **199,** 625–632 (2009).
2. Verkaik, N. J. *et al.* Immune evasion cluster-positive bacteriophages are highly prevalent among human *Staphylococcus aureus* strains, but they are not essential in the first stages of nasal colonization. *Clin. Microbiol. Infec.* **17,** 343–348 (2011).

Supplementary Table 5. *S. aureus* genes identified in isolates from GPA patients and HC. The detection of antigen-encoding genes in *S. aureus* isolates from both GPA patients and HC was determined with the Clondiag *S. aureus* Genotyping Kit 2.0 following the manufacture's instructions and as previously described (Alere Technologies GmbH, Jena, Germany) [1,2]. The *S. aureus* microarray contains 336 DNA probes for the detection of genes encoding for species markers, resistance, adhesion factors and virulence. Selected genes are displayed in numbers and percentages for both the 75 *S. aureus* isolates from GPA patients and the 18 *S. aureus* isolates from HC.

Gene Name	No. positive GPA isolates	% positive GPA isolates	No. positive HC isolates	% positive HC isolates
clfA	75/75	100	18/18	100
fnbpA	75/75	100	18/18	100
fnbpB	66/75	88	11/18	61
sasG	52/75	70	7/18	39
clfB	75/75	100	18/18	100
sdrD	66/75	88	14/18	78
hla	72/75	96	18/18	100
lukE	57/75	76	8/18	44
lukF	75/75	100	18/18	100
lukS	75/75	100	18/18	100
scn	72/75	96	14/18	78
lukD	61/75	81	6/18	33
chp	31/75	41	12/18	67
hlb	66/75	88	17/18	94
nuc	75/75	100	18/18	100
PVL	0/75	0	0/18	0
eta	2/75	3	0/18	0
etb	0/75	0	0/18	0
sec	4/75	5	1/18	6
tst-1	4/75	5	8/18	44
protein A	75/75	100	18/18	100
ssl9	75/75	100	18/18	100
ssl10	75/75	100	18/18	100
ssl3	68/75	91	16/18	89
sej	3/75	4	0/18	0
seb	25/75	33	0/18	0
sea	48/75	64	5/18	28
ssl5	75/75	100	18/18	100
seh	4/75	5	0/18	0
ssl11	52/75	70	10/18	56
seg	18/75	24	12/18	67
see	0/75	0	0/18	0
seq	18/75	24	0/18	0
sed	18/75	24	0/18	0
ser	3/75	4	0/18	0
sem	18/75	24	13/18	72
sei	18/75	24	13/18	72
sen	18/75	24	13/18	72
seo	18/75	24	13/18	72

References

1. Monecke, S., Slickers, P. & Ehricht, R. Assignment of *Staphylococcus aureus* isolates to clonal complexes based on microarray analysis and pattern recognition. *FEMS Immunol. Med. Mic.* **53,** 237–251 (2008).
2. Monecke, S., Jatzwauk, L., Weber, S., Slickers, P. & Ehricht, R. DNA microarray-based genotyping of methicillin-resistant *Staphylococcus aureus* strains from Eastern Saxony. *Clin. Microbiol. Infec.* **14,** 534–545 (2008).
